# A Bayesian multi-proxy contribution to the socioeconomic, political, and cultural history of late medieval Capitanata (southern Italy)

**DOI:** 10.1038/s41598-023-30706-9

**Published:** 2023-03-11

**Authors:** Carlo Cocozza, Wolf-Rüdiger Teegen, Ilaria Vigliarolo, Pasquale Favia, Roberta Giuliani, Italo Maria Muntoni, Domenico Oione, Lukas Clemens, Marcus Groß, Patrick Roberts, Carmine Lubritto, Ricardo Fernandes

**Affiliations:** 1 Institut für Vor- und Frühgeschichtliche Archäologie und Provinzialrömische Archäologie, and ArchaeoBioCenter, Ludwig-Maximilians-Universität München, Geschwister-Scholl-Platz 1, 80539 Munchen, Germany; 2 Department of Archaeology, Max Planck Institute of Geoanthropology, Kahlaische Str. 10, 07745 Jena, Germany; 3 Dipartimento di Scienze e Tecnologie Ambientali Biologiche e Farmaceutiche (DiSTABiF), and Mediterranean bioArchaeological Research Advances (MAReA) centre, Università degli studi della Campania “Luigi Vanvitelli”, Via Vivaldi 43, 81100 Caserta, Italy; 4 Dipartimento di Studi Umanistici, Università di Foggia, Via Arpi 176, 71121 Foggia, Italy; 5 Dipartimento di Ricerca e Innovazione Umanistica, Università degli Studi di Bari “Aldo Moro”, Strada della Torretta, 70122 Bari, Italy; 6 Soprintendenza Archeologia, Belle Arti e Paesaggio per le Province di Barletta-Andria-Trani e Foggia, Via Alberto Alvarez Valentini 8, 71121 Foggia, Italy; 7 Fachbereich Geschichte, Universität Trier, Universitätsring 15, 54296 Trier, Germany; 8 isoTROPIC Research Group, Max Planck Institute of Geoanthropology, Jena, 07745 Germany; 9 Climate Change and History Research Initiative, Princeton University, Princeton, USA; 10 Arne Faculty of Arts, Masaryk University, Nováka 1, 602 00 Brno, Czech Republic

**Keywords:** Biogeochemistry, Environmental sciences

## Abstract

Medieval southern Italy is typically viewed as a region where political, religious, and cultural systems coexisted and clashed. Written sources often focus on elites and give an image of a hierarchical feudal society supported by a farming economy. We undertook an interdisciplinary study combining historical and archaeological evidence with Bayesian modelling of multi-isotope data from human (n = 134) and faunal (n = 21) skeletal remains to inform on the socioeconomic organisation, cultural practices, and demographics of medieval communities in Capitanata (southern Italy). Isotopic results show significant dietary differences within local populations supportive of marked socioeconomic hierarchies. Bayesian dietary modelling suggested that cereal production, followed by animal management practices, was the economic basis of the region. However, minor consumption of marine fish, potentially associated with Christian practices, revealed intra-regional trade. At the site of Tertiveri, isotope-based clustering and Bayesian spatial modelling identified migrant individuals likely from the Alpine region plus one Muslim individual from the Mediterranean coastline. Our results align with the prevailing image of Medieval southern Italy but they also showcase how Bayesian methods and multi-isotope data can be used to directly inform on the history of local communities and of the legacy that these left.

## Introduction

The Middle Ages in Italy has been argued to have been marked by political and cultural fragmentation from the collapse of the western Roman empire (476 CE) until the end of the twelfth century, although other scholars note a persistence and reconfiguration of economic and urban networks^1–4^. Southern Italy represents a particularly interesting case of how populations in this part of the world responded to the collapse of the western Roman empire and transitioned into the new economic, social and cultural world of the medieval period^2,4,5^. Showing the strikingly high level of political fragmentation of the region, unlanded aristocrats from Normandy (France) famously sought their fortune in southern Italy during the eleventh century, intervening in local disputes among Lombard princes, Byzantine rulers, the Pope and Muslim pirates^4,6,7^. Through this, Normans accumulated wealth, land and titles culminating in the establishment of the Kingdom of Sicily in 1130 by the Hauteville family. The Normans imposed a feudal system based on their French experience, although it also included political traits from previous local Byzantine and Muslim forms of government. The Hauteville family was succeeded by the Swabian Hohenstaufen dynasty in 1194, which lasted until 1266, when Charles count of Anjou conquered the kingdom with papal support. While much is known historically about these political upheavals, far less is known in relation to the lived experiences of the multicultural populations navigating this changing social and economic landscape that was to reshape power dynamics in the Mediterranean world. Sites from southern Italy hence provide ideal case studies for exploring how changing political regimes and contact with areas of the eastern Roman empire, Mediterranean islands, and northern Africa altered economic systems and the demographics of local populations^2–4,8–10^. Furthermore, they were sites of increasing social differentiation during the medieval period that increasingly revolved around connection to the Christian faith and land ownership^7,10,11^.

Medieval Capitanata—a region in northern Apulia—has a centuries-long history as a buffer zone between Lombard states and Byzantine Apulia. However, the Norman conquest of the region in the mid-eleventh century led to its pacification and to wide economic transformations^12–14^. This pushed towards a demographic increase and to new food production systems adapted to local geographic features (e.g. fishing in coastal sites, pig husbandry in the plain and sheep/goat husbandry in the Apennines)^12^. Socio-economic shifts have been inferred through the analysis of the archaeological record, zooarchaeological studies, and limited archaeobotanical evidence on the region^12,13,15,16^. However, there is little anthropological^17^ and no biomolecular evidence for late medieval Capitanata. Previous archaeological isotopic studies in the region investigated only the Neolithic period^18–20^. The closest isotopic studies dating to the medieval period are those from Montella (Campania), Apigliano, and Quattro Macine (southern Apulia)^21,22^.

**Figure 1:**
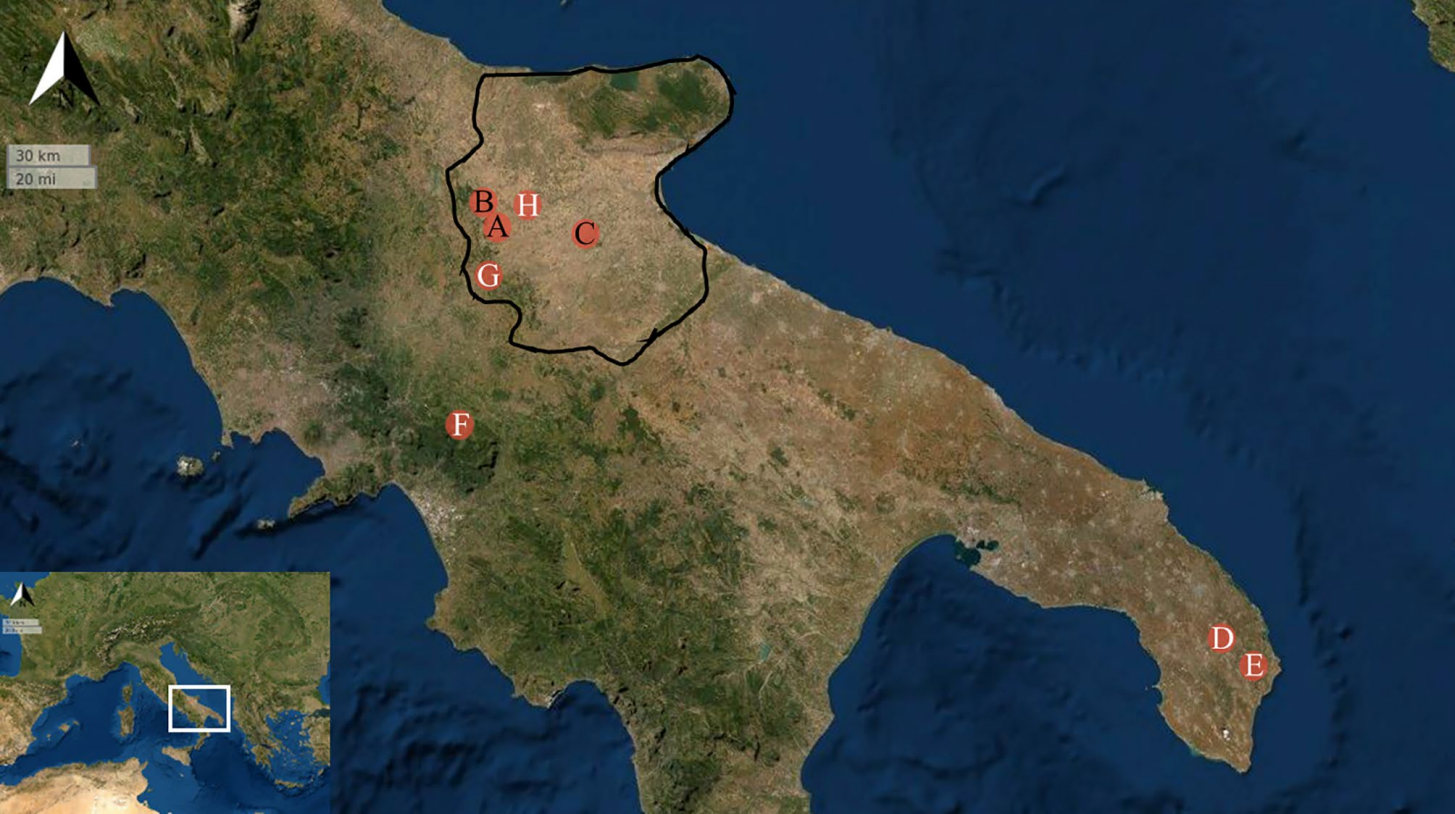
Spatial distribution of medieval sites from the historical region of Capitanata (border division in black) and southern Italy mentioned in this study: (**A**) Tertiveri; (**B**) Montecorvino; (**C**) San Lorenzo in Carminiano; (**D**) Apigliano; (**E**) Quattro Macine; (**F**) Franciscan Friary in Montella; (**G**) Crepacore Castle; (**H**) Lucera. With a black font, sites for which new isotopic data is presented. This map was generated using the Interactive map option in IsoMemoApp (https://isomemoapp.com/app/iso-memo-app, Data Search and Spatiotemporal modelling V. 22.12.3.1).

Overall, the Norman productive system was maintained for the entirety of the late medieval period, with a successive increase of agricultural production and the introduction of ‘*masserie*’^12,15^. These were large land estates often under direct administration of the crown. However, in the thirteenth century this region also observed the arrival of a melting-pot of cultures from both Mediterranean and continental regions. This is, for example, the case of Lucera (Fig. 1), a city that hosted the Islamic population that was deported from Sicily by order of the Hohenstaufen king Frederik II^4,23–25^. Tertiveri, one of the sites analysed in this study (Fig. 1), was also donated by the Angevin king Charles II to ‘Abd al- ‘Azīz, a powerful Muslim knight from Lucera^23,24^. Two individuals found in this site (here sampled and analysed) were buried according to an Islamic rite. Frederik II was also Holy Roman Emperor, but he kept his court in southern Italy and spent most of his time in Apulia^25^. Hence, diplomats, noblemen, and ecclesiastic personalities were often supposed to travel from northern Italy, Austria, and Germany to Capitanata. Additional evidence of the high-level of cultural admixture involves movements from Provence (France) during the late thirteenth century. Written records attest that the new Angevin king Charles I relocated soldiers to the turbulent Capitanata region to ensure its pacification^26^. This was followed by donations of lands and the settling of many Provençal individuals with their families^20^. Thus, late medieval Capitanata presents a high research potential to investigate the impact of migrations and economic systems on past societies.

Much of our historical knowledge of medieval southern Italy relies on written sources which often are not representative of a large proportion of past communities, particularly for population segments with a lower socioeconomic status^27^. Stable isotope analysis, by contrast, has been widely employed in archaeology to reconstruct past human subsistence or spatial mobility, and crop and animal management practices^21,22,28–31^. Isotopic ratios can be measured in osteological remains and, depending on the type of tissue analysed (e.g. collagen extracted from bones), information can be obtained for different periods of an individual’s lifetime^32–34^. The most common isotopic proxies employed in archaeological dietary studies are carbon (δ^13^C) and nitrogen (δ^15^N) isotopes measured in collagen extracted from bone or dentine. These are primarily informative on dietary protein consumption^35,36^, whereas δ^13^C measurements on carbonate from bone or tooth bioapatite inform on the dietary mix of protein, carbohydrates, and lipids. Measurement of the latter will more easily reveal the consumption of C_4_ plants when the primary source of protein is C_3_ (or vice-versa). Local environmental and climatic conditions determine the isotopic values of plant and animal food sources^37,38^. These can also be influenced by agricultural management practices such as irrigation, manuring, penning, etc.^36,39,40^. Thus, human isotopic values reflect the isotopic values of the food sources and their relative consumption. Human and animal spatial mobility can be investigated using δ^18^O measurements on carbonate from tooth enamel or bone bioapatite, taking into account potential complications caused by cooking activities, diagenesis and mathematical conversions of human δ^18^O values into δ^18^O of ingested water^41–44^.

We combined historical and archaeological evidence with novel Bayesian modelling of multi-isotope data (δ^13^C, δ^15^N, δ^18^O) from human (n = 134) and animal (n = 21) skeletal remains to inform on the socioeconomic organisation, cultural practices, and demographics of the medieval communities of Tertiveri, Montecorvino, and San Lorenzo in Carminiano (Capitanata, southern Italy). We were specifically interested in evaluating the implementation of a feudal system at the study sites. Individual isotopic variability was employed as a proxy for socioeconomic hierarchy and/or Christian religious practices. While overall population diets informed on the economic importance of farming and trade activities. The significance of external influences was investigated isotopically through the identification of migrants and the tracking of their places of origin.

## Results

Isotopic results, together with other supporting measurement, archaeological, and historical information are given in Supplementary Information file S1 and also deposited in the MATILDA online repository (https://www.doi.org/10.48493/w01v-fe90). The data was also aggregated to the CIMA database that compiles isotopic measurements for medieval archaeological samples^45,46^.

Bone and tooth samples (human = 134; fauna = 21) areoverall well-preserved in accordance with established parameters for bone collagen preservation^47^. However, fifteen human samples from Tertiveri show poor preservation with atomic C/N ratios outside the acceptable range. In addition, sample TC100 did not produce sufficient collagen for measurement. These represent 11.9% of the human dataset. Bone carbonate results for samples with bad collagen preservation were also excluded from our analysis^48^.

### Faunal isotopic results

**Figure 2:**
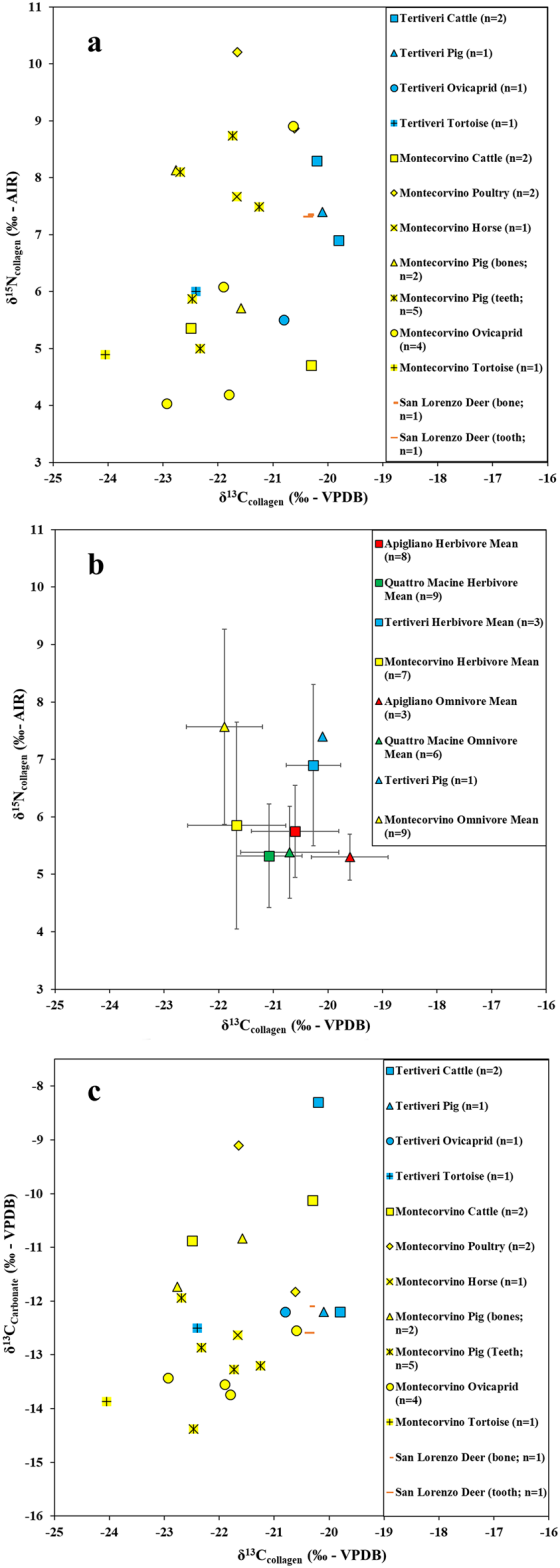
Isotopic results for archaeological faunal samples. (**a**) δ^13^C versus δ^15^N for fauna from this study; (**b**) δ^13^C versus δ^15^N comparing mean isotopic values for herbivores and omnivores from this study versus those measured in the coeval Apulian sites of Apigliano and Quattro Macine^22^; (**c**) δ^13^C_collagen_ versus δ^13^C_carbonate_ for fauna from this study.

Faunal bulk bone and tooth collagen δ^13^C and δ^15^N measurements range from −24.1 to −19.8‰ and from 4.7 to 10.1‰, respectively (Fig. 2a). For δ^13^C, there is a significant difference between the Montecorvino and Tertiveri animals (mean δ^13^C: −21.9 ± 0.9‰; −20.7 ± 1.0‰). The sample size is small making the interpretation of the results difficult, but the difference is likely associated with differences in local environmental conditions and/or animal management practices, including a possible ‘canopy effect’ associated with dense forests surrounding Montecorvino^40,49^. These are mentioned in Angevin documents^50^ and also attested by palaeo-environmental analysis^51^. For δ^15^N values there are no significant differences between the two sites (mean: 6.7 ± 1.9‰ for Montecorvino; 6.8 ± 1.1‰ for Tertiveri). However, the standard deviations for Montecorvino pigs and sheep/goats are relatively large (7.0 ± 1.5‰; 5.8 ± 2.3‰, respectively) when compared to δ^15^N values measured in the same taxa from two other late medieval sites in southern Apulia, i.e. Apigliano (pigs: 5.3 ± 0.4‰; sheep/goat: 6.1 ± 0.4‰) and Quattro Macine (pigs: 5.8 ± 0.7‰; sheep/goat: 5.4 ± 0.8‰)^23^ (Fig. 2b). Overall, domestic herbivores from Capitanata display larger δ^15^N ranges (4.0–8.9‰; mean: 6.2 ± 1.7‰) in comparison to those from southern Apulia (4.1–6.9‰; mean: 5.5 ± 0.9‰). These likely reflect a variety of animal husbandry strategies including intensive husbandry, penning, feeding with plants grown in fields subject to manuring, and transhumance^39,40,52^.

The deer remains from San Lorenzo (SLDE1) have a high δ^15^N collagen value (bone: 7.4‰; tooth: 7.3‰. Figure 2a) when compared to values observed for modern specimens^53^. Given historical evidence for a royal hunting ground around San Lorenzo in Carminiano^54^, it is possible that deers had their diet managed by humans, which may have included access to ^15^N-enriched plants. The bone remains of two tortoises from Tertiveri (δ^13^C: −22.4‰; δ^15^N: 6.0‰) and Montecorvino (δ^13^C: −24.1‰; δ^15^N: 4.9‰) have low δ^13^C values suggesting that they fed on freshwater sources^55^. Tortoises were commonly consumed in medieval Italy and zooarchaeological remains are often found in sites associated with the clergy, since they were considered to be ‘fish’ and not included in the list of proscribed foods by medieval religious fasting rules^9,56^.

Results of stable carbon isotopes for bone bioapatite in animals range between −14.1‰ and −8.3‰ (mean: −12.1 ± 1.5‰) (Fig. 2c). The highest values for animals are observed for a chicken from Montecorvino (MOCH2 δ^13^C_carbonate_: −9.1‰; δ^13^C_collagen_: −21.7‰; δ^15^N: 10.2‰) and a cattle specimen from Tertiveri (TCCA2 δ^13^C_carbonate_: −8.3‰; δ^13^C_collagen_: −20.2‰; δ^15^N: 8.3‰), which were likely fed with a higher contribution of C_4_ cereals compared to other animals. However, δ^13^C_collagen_ values from these animals clearly reflect C_3_ dominated diets. In the case of the chicken (MOCH2), the isotopic difference between δ^13^C_bioapatite_ and δ^13^C_collagen_ signals could derive from the combination of a predominant C_3_ protein source and an overall caloric contribution that included significant C_4_ components. Such an explanation is more difficult to extend to the cattle specimen (TCCA2), since this animal would only consume plants. A mixed diet of legumes and C_3_/C_4_ plants seems unlikely given that the animal has relatively high nitrogen values. Even assuming that this cattle specimen consumed plants from manured fields, which elevates plant δ^15^N values, a predominant protein source from legumes (typically near 0‰ δ^15^N values) would be inconsistent with our measurements. Another possibility is that the bioapatite and collagen δ^13^C signals reflect different lifetime dietary stages. This would imply that bone collagen and bioapatite have different renewal rates as reported previously for other species^57^. However, these are unknown.

**Figure 3:**
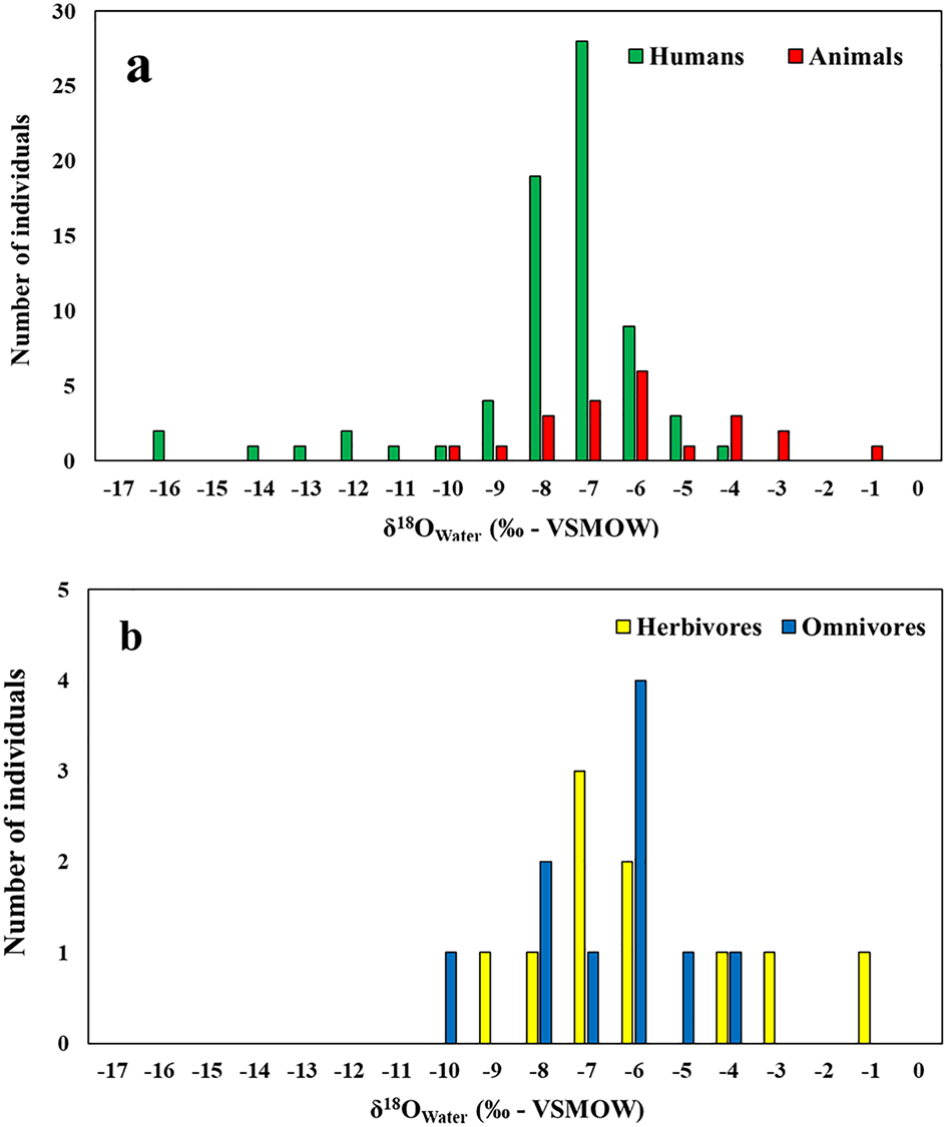
Calculated values for ingested water δ^18^O_Water_ for humans and fauna. (**a**) histogram showing the distribution of human and faunal δ^18^O_Water_ values; (**b**) histogram showing the distribution of domesticated herbivore and omnivore δ^18^O_Water_ values.

Values for bioapatite and enamel δ^18^O in fauna from Tertiveri and Montecorvino show a wide range (measured δ^18^O_carbonate_ range: −6.9‰ to −1.6‰; mean: −4.4 ± 1.3‰; calculated δ^18^O_water_ range: −10.4‰ to −1.7‰; mean: −6.3 ± 2.1‰) (Fig. 3a). The age and time of death of the animals or the renewal rates of skeletal material are not known. Thus, some variation may be explained by seasonal shifts in water δ^18^O values. For instance, pigs were commonly slaughtered at the end of the year, whereas sheep/goats were butchered around Easter in accordance with religious practices^9,56^. Spatial variations in water δ^18^O values may have also had an impact, since vertical transhumance of ovicaprids and cattle has been recorded for late medieval Capitanata^58,59^. Although there is a large overlap between the δ^18^O values for domesticated herbivores and omnivores (Fig. 3b).

One sheep/goat bone from Montecorvino (MOSG1) shows an unusually high δ^18^O_water_ (−1.7‰) value. In addition to spatial and temporal variations in water δ^18^O values it is possible that this sheep was consuming leaf water with δ^18^O values higher than meteoritic water^60^. Ovicaprids are semi-obligate drinkers and obtain most of the water through the plants they consume^61^. Other sheep/goats from Montecorvino have δ^18^O_water_ values between −6.1 and −4.0‰. However, one sheep/goat from Tertiveri (TCSG1) has the lowest δ^18^O_water_ value (−7.6‰) among ovicaprids. Multiple factors could explain such variability. Differences in feeding behaviours, including the aforementioned consumption of leaf water, the practice of transhumance at various spatial and temporal scales, plus differences in killing season could explain the wide observed range in δ^18^O_water_ values. To resolve this, it is necessary to study animal movements and diets at higher temporal resolutions by undertaking future isotopic measurements on tooth sections^61,62^.

### Human isotopic results and Bayesian modelling

**Figure 4:**
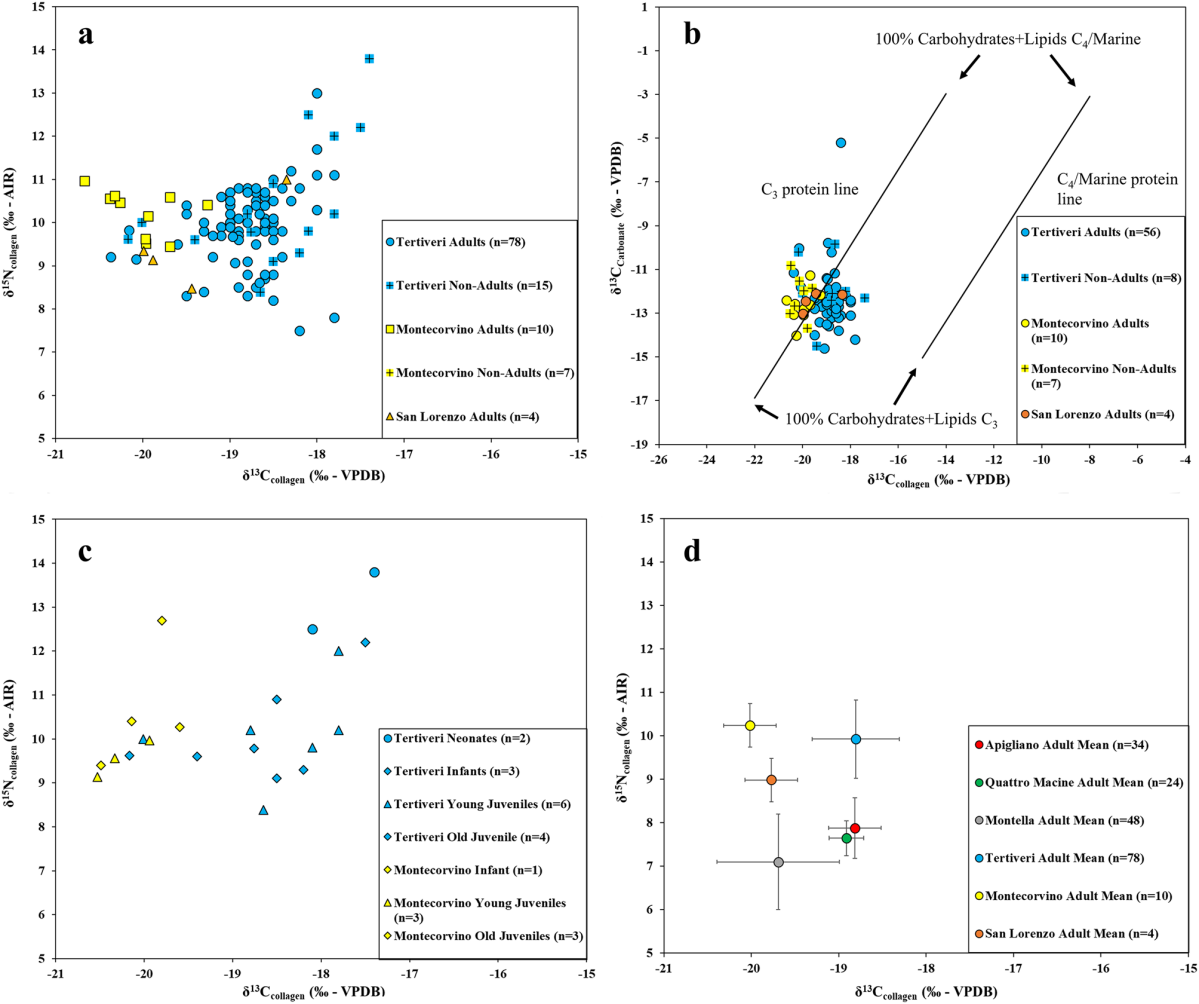
Human isotopic results. (**a**) δ^13^C_collagen_ versus δ^15^N_collagen_ values for human individuals from this study; (**b**) δ^13^C_collagen_ versus δ^13^C_carbonate_ values for human individuals from this study (reference dietary lines after Kellner and Schoeninger)^65^. (**c**) δ^13^C_collagen_ versus δ^15^N_collagen_ values for human non-adult individuals from this study. Neonates: 0–1 y.; Infants: 1–3 y.; Young Juveniles: 3–7 y.; Old Juveniles: 7–13 y. (**d**) δ^13^C_collagen_ versus δ^15^N_collagen_ biplot comparing mean isotopic values for adults from this study with those measured in the coeval southern Italian sites of Montella, Apigliano, and Quattro Macine (Torino et al*.* 2015; Rolandsen et al*.* 2019)^21,22^.

Results for stable carbon and nitrogen isotopic values measured in human bone collagen from the region of Capitanata range from −20.7 to −17.4‰ and from 7.5 to 13.8‰, respectively (Fig. 4a). Mean values are −19.0 ± 0.7‰ for δ^13^C and 10.0 ± 1.0‰ for δ^15^N. Human δ^13^C results measured in bone carbonate range from −14.6 to −5.2‰ (mean: −12.3 ± 1.2‰) (Fig. 4b). Isotopic results for non-adults are presented separately from those of adolescents and adults. Non-adult individuals may include a dietary isotopic signal from the consumption from human milk and/or from food employed during infancy in post-weaning strategies^63,64^. Figure 4c demonstrates the distribution of δ^13^C and δ^15^N values for non-adults in Tertiveri (mean δ^13^C_collagen_: −18.5 ± 0.8‰; mean δ^15^N: 10.5 ± 1.5‰; δ^13^C_carbonate_: −11.9 ± 1.4‰) and Montecorvino (mean δ^13^C_collagen_: −20.1 ± 0.4‰; mean δ^15^N: 10.2 ± 1.2‰; δ^13^C_carbonate_: −12.2 ± 1.0‰). There is no significant difference among age groups for non-adults (Fig. 4c), although neonates present higher δ^15^N values, which is likely a result of breastfeeding. From San Lorenzo only four adult individuals were analysed (mean δ^13^C_collagen_: −19.8 ± 0.3‰; mean δ^15^N: 9.0 ± 0.5‰; δ^13^C_carbonate_: −12.4 ± 0.4‰), while from Tertiveri 78 adults present an acceptable level of bone preservation (mean δ^13^C_collagen_: −18.8 ± 0.5‰; mean δ^15^N: 9.9 ± 0.9‰; δ^13^C_carbonate_: −12.4 ± 1.3‰) and from Montecorvino tenadults were analysed (mean δ^13^C_collagen_: −20.0 ± 0.4‰; mean δ^15^N: 10.2 ± 0.5‰; δ^13^C_carbonate_: −12.7 ± 0.7‰).

The δ^13^C_collagen_ values for Tertiveri are significantly higher than Montecorvino (Mann–Whitney U test: U = 26, n1 = 78, n2 = 10, P < 0.05, two-tailed) and this may be related to differences in the isotopic baseline (e.g. environmental factors such as the ‘canopy effect’ or specific animal management practices such as C_3_/C_4_ feeding selection) or to differences in human diets. No significant stable carbon and nitrogen isotopic differences between adult male (mean δ^13^C: −18.7 ± 0.4‰; mean δ^15^N: 10.0 ± 0.9‰) and female (mean δ^13^C: −18.8 ± 0.7‰; mean δ^15^N: 10.0 ± 1.2‰) individuals were detected at Tertiveri (δ^13^C: Mann–Whitney U test: U = 800.5, n1 = 36, n2 = 17, P > 0.05, two-tailed; δ^15^N: Mann–Whitney U test: U = 807.5, n1 = 36, n2 = 17, P > 0.05, two-tailed), whereas at Montecorvino and San Lorenzo the small sample size does not allow for comparisons. When compared with previously published studies for medieval southern Italy, adult individuals from Capitanata show overall higher δ^15^N values than Montella (mean δ^13^C: −19.7 ± 0.7‰; mean δ^15^N: 7.1 ± 1.1‰), Apigliano (mean δ^13^C: −18.8 ± 0.3‰; mean δ^15^N: 7.9 ± 0.7‰), and Quattro Macine (mean δ^13^C: −18.9 ± 0.2‰; mean δ^15^N: 7.6 ± 0.4‰) (Fig. 4d)^21,22^. The δ^15^N values measured in faunal specimens from Capitanata were also higher (Fig. 2b), implying that the isotopic baseline, rather than diet, is the primary factor that accounts for the isotopic difference in adult populations from different sites.

The results for δ^13^C_carbonate_ *versus* δ^13^C_collagen_ are plotted in Fig. 4b and compared to C_3_ protein and C_4_/marine protein reference lines after Kellner and Schoeninger^65^. This shows that C_3_ foods were the main protein source although there were minor contributions from C_4_/marine protein foods for several individuals. The contribution from C_4_ carbohydrates is also noticeable for several individuals having relatively high δ^13^C_carbonate_ values. One individual (TC65) has particularly high δ^13^C_carbonate_ values (δ^13^C_carbonate_: −5.2‰; δ^13^C_collagen_: −18.4‰; δ^15^N: 10.8‰) suggesting a large consumption of C_4_ cereals. The source of protein for this individual was predominantly C_3_ although there could have been a minor contribution from marine resources.

**Figure 5:**
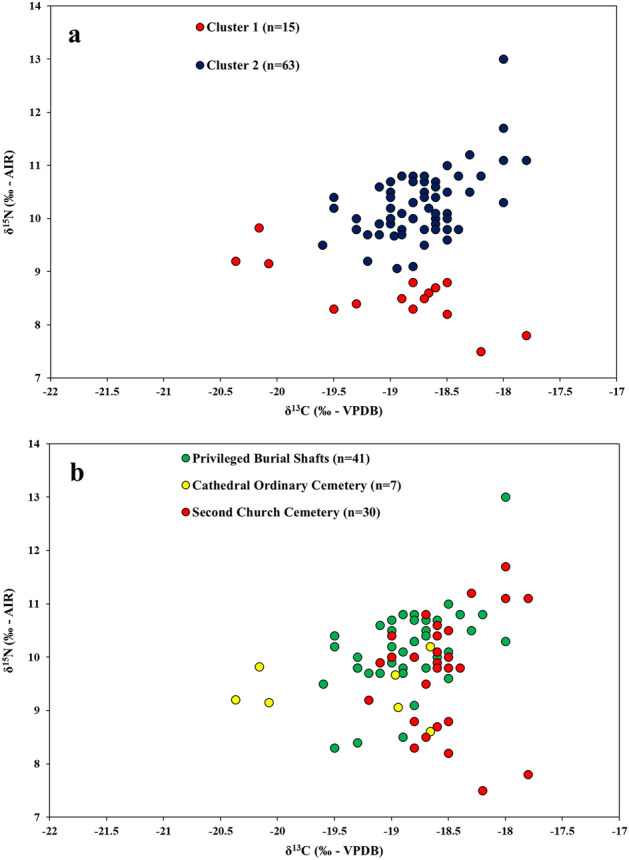
Distribution of δ^13^C_collagen_ and δ^15^N_collagen_ values for human adult individuals from Tertiveri. (**a**) Cluster classification; (**b**) Archaeological classification.

Cluster analysis of adult human δ^13^C_collagen_ and δ^15^N_collagen_ values from Tertiveri revealed two main data clusters (details in "Methods" section) (Fig. 5a). Cluster 1 shows a negative correlation between δ^13^C_collagen_ and δ^15^N_collagen_ values suggesting a mixing line from C_3_ to C_4_ protein consumption. In contrast, Cluster 2 shows a roughly positive correlation between δ^13^C_collagen_ and δ^15^N_collagen_ values suggesting a mixing line from C_3_ protein (lower isotopic values) to marine protein (higher isotopic values) consumption. The former would include C_3_ animal protein with δ^15^N values higher than C_4_ plants (e.g. millet).

**Figure 6:**
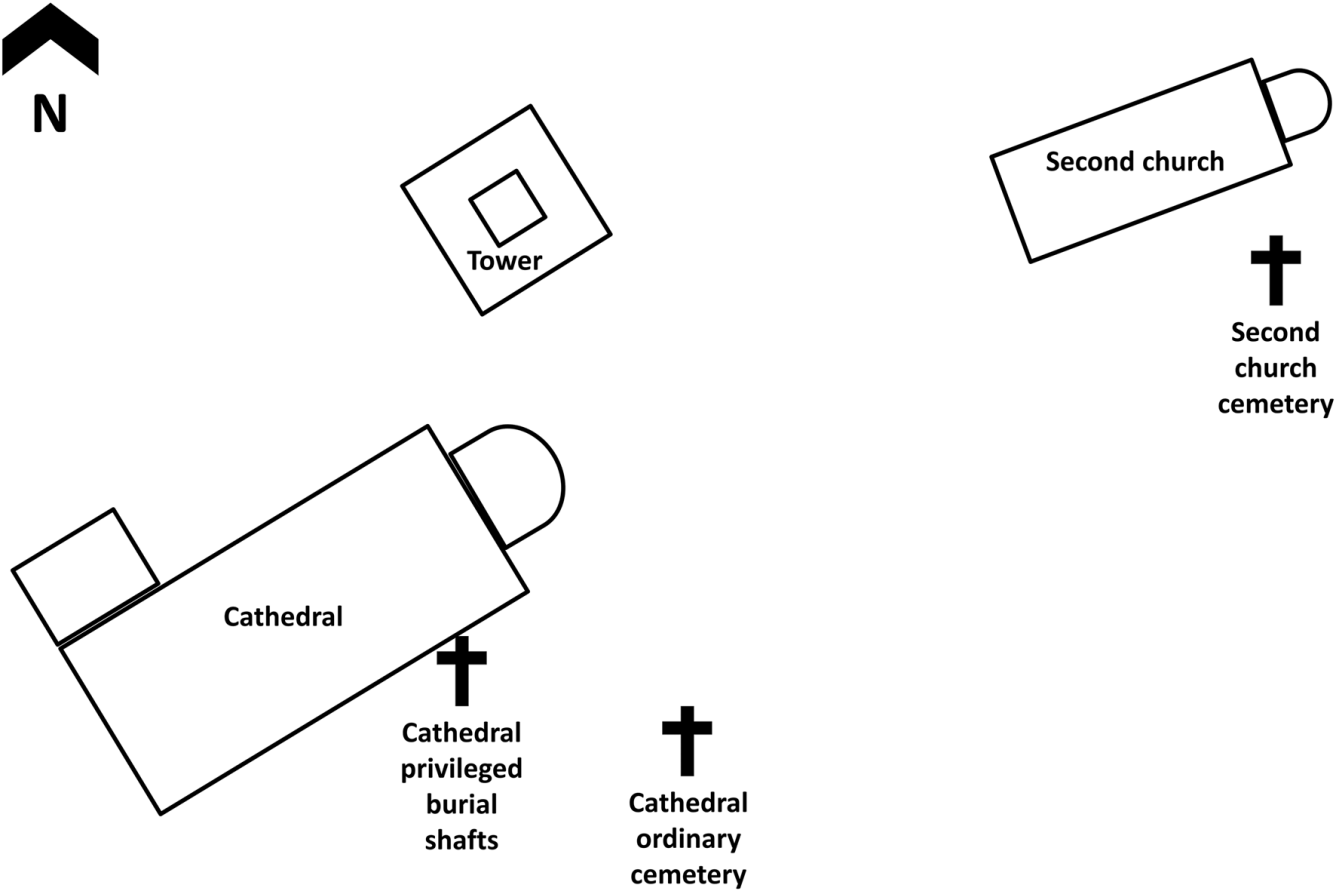
Schematic representation of the relative positions of burial locations at Tertiveri. The figure is not to scale.

A different classification of human δ^13^C_collagen_ and δ^15^N_collagen_ values is also offered by archaeological descriptions. This is shown in Fig. 5b (burial locations are outlined in Fig. 6 and more details are given in Supplementary Info File S2). A privileged status is inferred for adult individuals buried in shafts discovered outside the Cathedral and parallel to the lateral walls (n = 41; mean δ^13^C_collagen_: −18.9 ± 0.4‰; mean δ^15^N: 10.1 ± 0.8‰; δ^13^C_carbonate_: −12.5 ± 1.4‰). A group of individuals were also recovered outside the remains of a second church reflecting a wider social stratification (n = 30; mean δ^13^C_collagen_: −18.6 ± 0.4‰; mean δ^15^N: 9.7 ± 1.0‰; δ^13^C_carbonate_: −12.5 ± 0.9‰). Finally, individuals were also discovered in the so-called ‘ordinary’ cemetery around the Cathedral, but only the collagen extracted from seven individuals were sufficiently well preserved for analysis (mean δ^13^C_collagen_: −19.4 ± 0.8‰; mean δ^15^N: 9.4 ± 0.5‰; δ^13^C_carbonate_: −10.9 ± 0.7‰). When the two classifications (i.e. isotopic and archaeological) are compared, we notice that: for Cluster 1 (n = 15) 20% of its individuals are from privileged shafts; 53% from outside a second church; and 27% from the ‘ordinary’ cemetery located outside the Cathedral. Whereas these percentages for Cluster 2 (n = 63) are 60%, 35%, and 5%, respectively. Overall, results suggest a higher consumption of marine resources by higher status individuals whereas lower status individuals would consume a higher proportion of C_4_ plants, likely millet.

For dietary quantitative estimation, we employed the Bayesian mixing model ReSources (an updated version of FRUITS^66^, details on model settings can be found in Supplementary Information File S3). Seven main food groups were considered for dietary reconstruction (C_3_ plants, C_4_ cereals, cattle, ovicaprid, pigs, poultry, and marine resources). Additional Bayesian priors were included as constraints to improve dietary resolution. These were grounded on evidence obtained from archaeofaunal, archaeobotanical, and ethnographic studies plus from written records (Supplementary Information File S3). However, a relatively minor contribution from other resources (e.g. freshwater fish) cannot be excluded.

**Figure 7:**
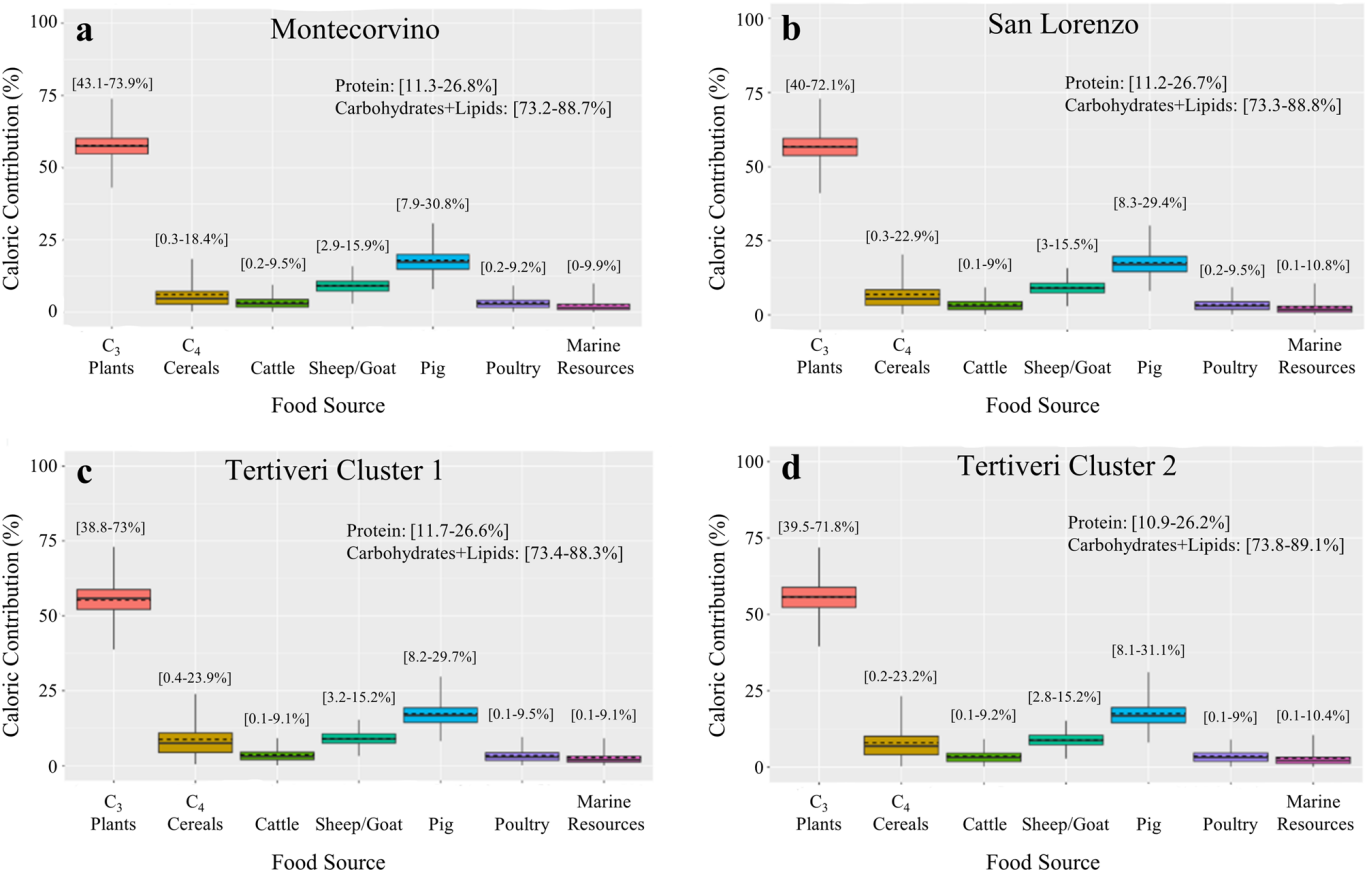
Bayesian estimates of dietary caloric contributions (boxes and whiskers represent 68% and 95% credible ranges, respectively. Horizontal continuous lines represent the mean and dashed horizontal lines the median). Numbers in brackets represent 95% credible ranges. Also included within the graph are numeric estimates of the caloric contributions of protein *versus*. carbohydrates macronutrients. Estimate for: (**a**) Montecorvino; (**b**) San Lorenzo in Carminiano; (**c**) Tertiveri Cluster 1; (**d**) Tertiveri Cluster 2.

Bayesian dietary caloric estimates are shown in figure Fig. 7. The magnitude of the uncertainties for these do not allow us to identify clear dietary differences among the sites. Nonetheless, the modelling shows that C_3_ plants (e.g. wheat, or barley, fruit, vegetables, legumes, and nuts) were the main source of calories for late medieval Capitanata. This is followed by pig products and by ovicaprid products (e.g. mutton, milk, cheese). Consumption of C_4_ cereals is possible for all sites although the wide credible ranges allow for a near zero dietary contribution. A small contribution from marine fish intake is possible which would indicate trade with coastal settlements. Cattle and poultry products are also estimated to be consumed in very small amounts.

Human δ^18^O_carbonate_ values range from −13 to −3.2‰ (mean: −5.8 ± 1.6‰) (calculated δ^18^O_water_ range: −16.5 to −5.3; mean: −8.4 ± 2.2‰, Fig. 3a). Given the potential for a nursing signal in infants, in the following discussion we only consider adult individuals from Tertiveri and Montecorvino which given their proximity would likely have similar δ^18^O_water_ values. For San Lorenzo only four individuals were available for analyses and the site is located at a greater distance from Tertiveri and Montecorvino.

**Figure 8:**
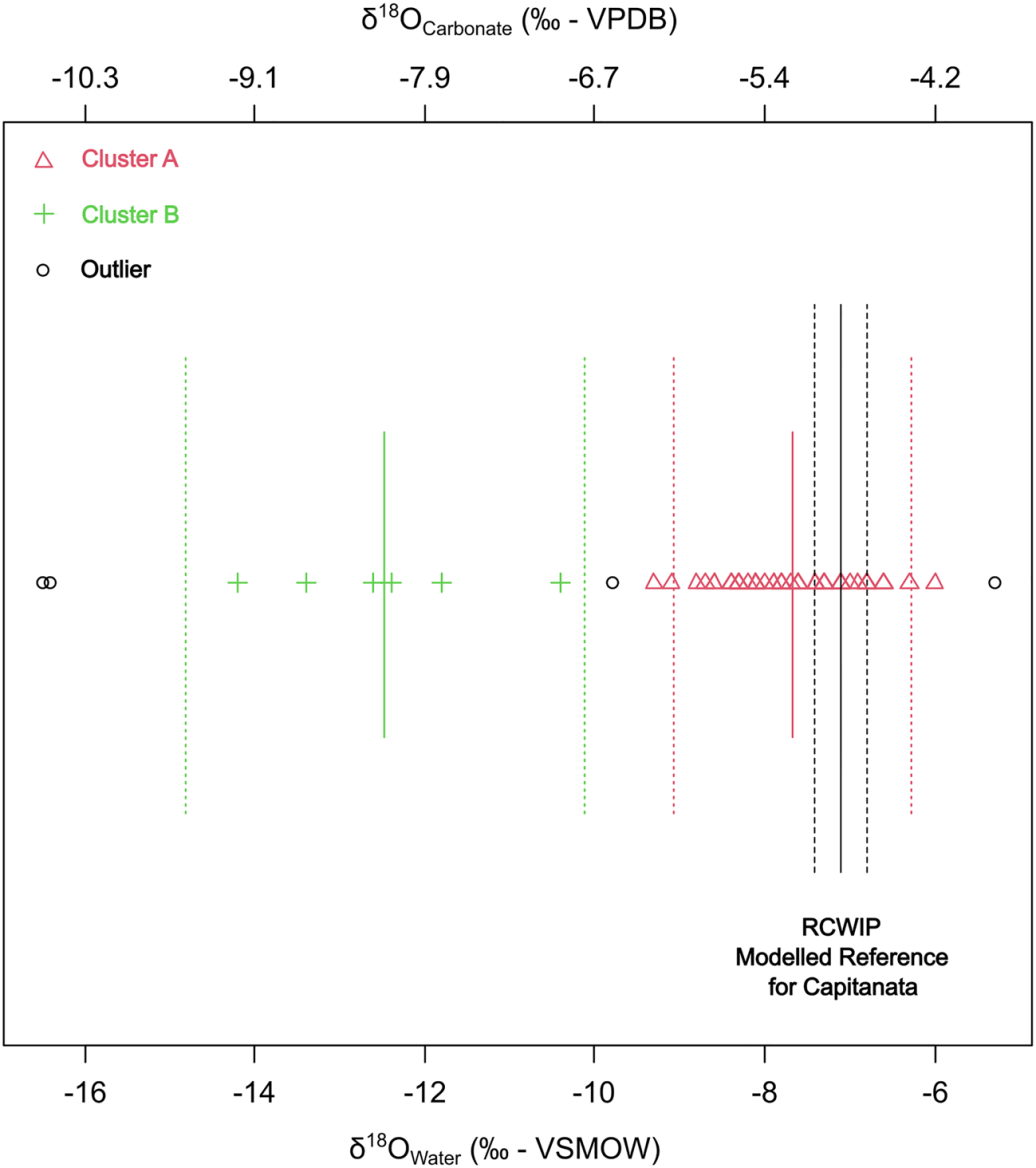
Cluster classification and outlier detection for the distribution of δ^18^O values for adult individuals from Tertiveri and Montecorvino. Solid green and red lines represent cluster mean values, whereas green and red pointed lines represent a 2-sigma interval. Continuous vertical black line represents the mean δ^18^O_Water_ value for Tertiveri and Montercorvino ( −7.1‰) determined using Bayesian modelling of RCWIP estimates while dashed vertical black lines represent the standard error of the mean for the estimates (0.3‰) (Terzer et al. 2013)^67^.

**Figure 9:**
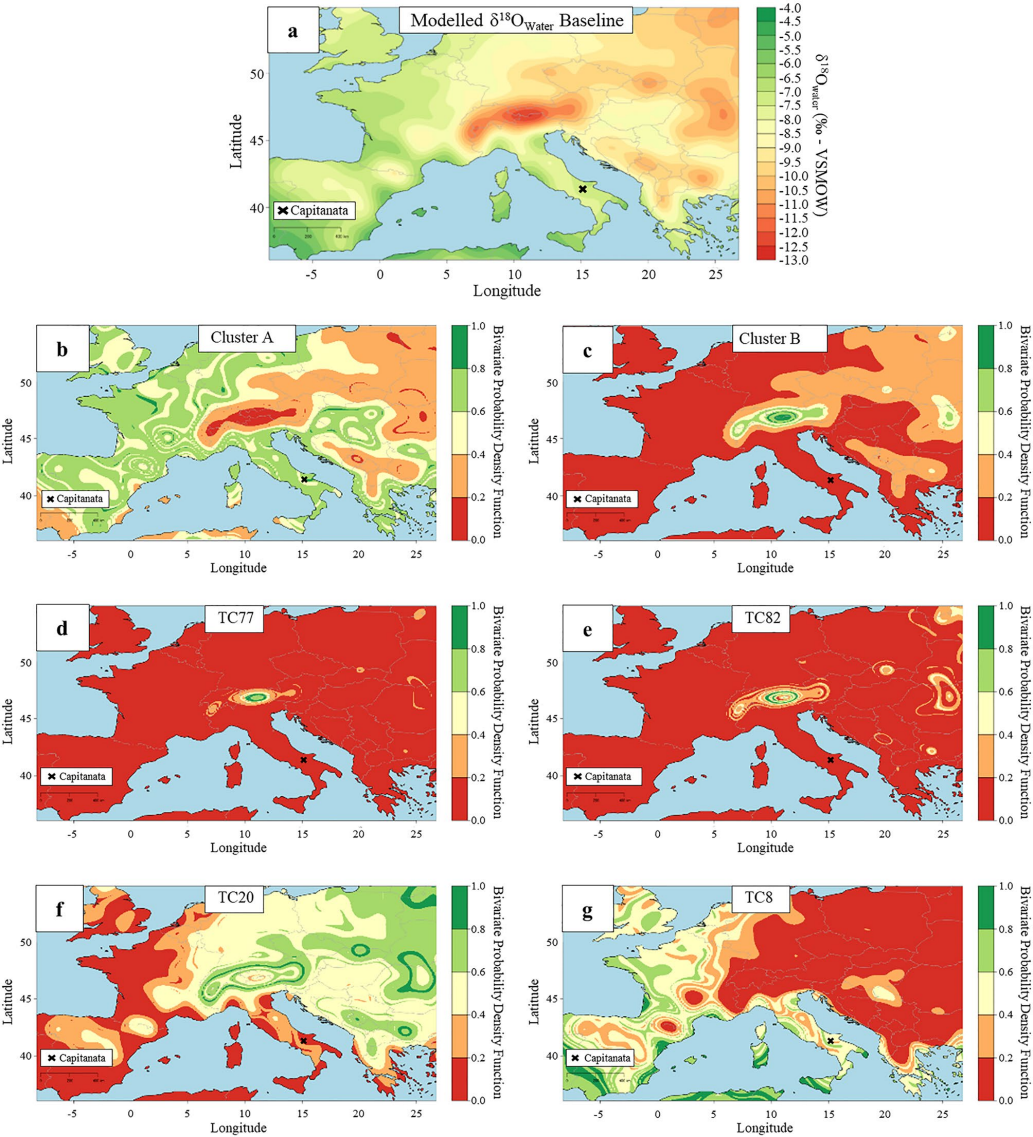
Residence probability maps assigned using the model LocateR. (**a**) Bayesian modelled δ^18^O_water_ isotopic baseline based on RCWIP model. (**b**) Cluster A (mean value); (**c**) Cluster B (mean value); (**d**) TC77; (**e**) TC82; (**f**) TC20; (**g**) TC8. Maps were generated using AverageR and LocateR modelling options from IsoMemoApp (https://isomemoapp.com/app/iso-memo-app, Data Search and Spatiotemporal modelling V. 22.12.3.1). More details in the "Methods" section.

Cluster classification with outlier detection (threshold 5%) was employed to classify the δ^18^O values for Tertiveri and Montecorvino adult individuals (see "Methods" section) (Fig. 8). Two clusters and four outliers were identified. A total of 57 individuals were assigned to Cluster A (δ^18^O_water_ range: −9.8‰ to −6‰; mean: −7.7 ± 0.8‰), whereas five individuals were assigned to Cluster B (δ^18^O_water_ range: −14.2‰ to −11.8‰; mean: −12.9 ± 0.9‰). The distribution of calculated human δ^18^O_water_ values was compared to local water estimates based on the point values from the Regionalized Cluster-based Water Isotope Prediction model (RCWIP)^67^. Using these, a Bayesian reference baseline for δ^18^O_Water_ values across western Europe was generated and the local average for Tertiveri and Montecorvino was estimated (Fig. 9a). This value ( −7.1‰ for a standard error of the mean of 0.3‰) is close to the δ^18^O_water_ average for Cluster A which is assumed to represent individuals residing in Tertiveri or Montecorvino. In contrast Cluster B represents incoming individuals. The overall variability for each cluster can be explained by post-mortem alterations to in vivo bone δ^18^O values due to diagenesis or to changes to local water δ^18^O values due to cooking/brewing practices^41,42,44^.

In addition to the above mentioned factors influencing ingested water and post-mortem bone δ^18^O values it is necessary to note that the bone bioapatite is undergoing renewal during the lifetime of an individual. Thus, the estimated human δ^18^O_Water_ values are potentially the result of mixed contributions from water sources having different δ^18^O_Water_ values. The outlier TC20 is placed between clusters A and B and could represent an individual originating from a location outside Tertiveri that moved and resided there for a considerable period of time. It is also possible that clusters A and B themselves include individuals with mixed δ^18^O values. In the case of cluster A this is another variable to consider on why the average for this cluster is slightly lower than the estimated local RCWIP value.

The model LocateR^68^ was employed to assign a spatial probability distribution for the place of residence associated with individuals from clusters A and B plus the four outliers. The model compares estimated human δ^18^O_Water_ values with the spatial distribution of RCWIP values (Fig. 9a). As expected for Cluster A there is a high residence probability assigned to the Tertiveri/Montecorvino region (Fig. 9b) whereas for Cluster B highest probabilities are observed for the alpine region (Fig. 9c).

The four outliers correspond to individuals buried at Tertiveri. Individual TC77 ( −16.4‰) and TC82 ( −16.5‰) have the lowest δ^18^O_Water_ values within the dataset and high residence probabilities assigned to the Alpine region (Fig. 9d,e). These individuals likely died within a short time of their arrival at Tertiveri. Individual TC20 was also assigned a high probability of residence in the Alpine region although high probabilities are also observed for eastern European regions (Fig. 9f). However, as mentioned above this individual is likely to have a strong mixed signal making the interpretation of LocateR results difficult. Individuals TC77, TC82, and TC20 were all found within the same shaft burial assumed to belong to privileged individuals. The fourth outlier, TC8 ( −5.3‰), corresponds to an adult male individual buried in accordance with an Islamic burial rite. His death likely took place in 1296–1300 CE, when Tertiveri was controlled by a Muslim knight^23,69,70^. For this individual LocateR shows high residence probabilities for the Mediterranean coastline, including the southern tips of the Italian peninsula and Sicily (Fig. 9g).

## Discussion

The main source of calories for humans across the three sites was C_3_ plants, a food group consisting of plants such as wheat, barley, rye, oat, fruit, nuts, vegetables, and legumes. Historical evidence suggests that these foods were the staple diet for southern Italian populations during the Middle Ages^9^. In Capitanata, the presence of multiple grain storage buildings (e.g. silos) marks the importance of cereal production as a main economic activity during the late Middle Ages^71^. However, these sources do not mention C_4_ cereals as a significant crop. In contrast, our isotope-based estimates revealed the potential intake of a moderate amount of C_4_ plants across the different sites. This is clearer for Tertiveri where individuals from Cluster 1 are in a mixing line between C_3_ and C_4_ protein (Fig. 5). The earliest consumption of C_4_ cereals in southern Italy (likely millet) was isotopically detected for the Bronze Age^72^, although C_3_ plants still dominated diets. A useful characteristic of C_4_ cereals is that these overall ripen faster than C_3_ cereals and are more durable^73^. Millet and sorghum are grown during the spring/early summer periods in southern Italy. This is when transhumance practices from Apulia to the Abruzzo Apennines took place^58,59^ and so it is possible that the cultivation of C_4_ crops in Capitanata may have been part of a transhumance economy, whereby shepherds consumed these cereals during their summer journeys across the Apennines.

Bayesian caloric estimates show that pigs were likely the main dietary source of animal products. This is unsurprising, given the accessible prices for pig products during the late Middle Ages in comparison to other animal meats^9,74^. A pig-based farming economy is often observed in association with demographic growth (e.g. imperial Rome^75^). Pig husbandry, paired with cereal production and transhumance, became the basis for the farming economy of late medieval Capitanata^76^. This matches the demographic growth following the onset of Norman rule which would require more intensive agricultural production^13^.

During the late medieval period there was an increase in transhumance in the region^59^. Tertiveri and Montecorvino were located in the vicinity of transhumance routes and as a likely result of more intense animal traffic the local consumption of sheep/goat products increased. Bayesian estimates confirm a significant dietary consumption of sheep/goat products, either as meat (mutton) or dairy products, whereas estimates for cattle consumption were small. However, it is known from historical sources that cattle transhumance was also practiced^58^. During the late medieval period, cattle were usually used for ploughing, whereas their meat had a high market value and was therefore mostly consumed from old animals which could no longer be employed in the fields^9,15,74^. Our modelling results also indicate that poultry meat and eggs consumption was overall probably small.

The caloric contribution from marine foods was small across the different sites. However, given the distance for investigated sites and the coast, this suggests the presence of intra-regional trade networks connecting Adriatic sites with the Apennines. In Tertiveri, individuals from Cluster 2 (Fig. 5a) had bone collagen isotopic values along a rough mixing line between C_3_ and marine protein. These contrast with individuals from Cluster 1 where the consumption of C_4_ cereals was higher although Bayesian estimates do not offer very precise estimates. What is clear is that there is a predominance of high socioeconomic individuals in Cluster 2, in contrast to Cluster 1. Millet was considered a low status food during antiquity while the fish imports were likely to be expensive and more easily accessible to individuals with a higher socioeconomic status^8,9^.

Included in Cluster 2 is the so-called Tertiveri bishop (TC74)^70^ showing moderately elevated isotopic values. The consumption of marine foods during the medieval period is often associated with the clergy, given that these represented one of the wealthier strata of society and were more likely to adhere to Catholic fasting rules^56^. Such rules varied with time, region and monastic order, but in the extreme could imply that terrestrial meat could not be consumed for up to nearly half of the year^56^. Whilst the so-called bishop does not have the highest isotopic values within the cluster, it is possible that the age of the individual (c. 70–80 years old) may have influenced dietary practices. Moreover, old people were often dispensed from religious fasting^56^.

Two Muslim individuals (TC8 and TC21) were also found in Tertiveri and show bone collagen isotopic values that do not suggest the consumption of marine foods. Islamic fasting rules forbid the consumption of pork, which within our dataset has distinct isotopic values relatively to the other terrestrial food sources. Individual TC21 is an infant (0–3 years) and their isotopic values likely include a nursing effect^63^. Individual TC8 is an adult with a diet mainly consisting of cereals (mostly C_3_ but with a moderate contribution from C_4_), legumes and ovicaprid products. It should be noticed, however, that from the observed bone collagen isotopic values for TC8 it is not possible to fully exclude pork consumption.

Previous isotopic results for late medieval southern Italy are only available for three sites, i.e. Apigliano and Quattro Macine in southern Apulia^22^ and the Franciscan monastery of Montella in the Campanian apennines^21^. The δ^15^N bone collagen values for the adult population from these sites are considerably lower than those presented in our study. This could be the result of dietary differences and/or differences in food values as a result of varying environmental conditions and farming practices^36–40^. Manuring can elevate the δ^15^N value of crops^39^. As mentioned above, the Capitanata region had an economy based on cereal cultivation plus the zooarchaeological record shows the presence of ovicaprids, cattle, and pigs making the practice of manuring possible. There are no direct isotopic measurements on plants for the studied region or from previous publications. However, herbivores from Capitanata show elevated δ^15^N values when compared to those from Apigliano and Quattro Macine (the site of Montella had no faunal remains). The same applies to pigs. Similar regional disparities in agricultural practices and environmental conditions were observed in the rest of Italy, Iberia, and England^45^. This is associated with a high degree of economic and/or political fragmentation in Europe during the Middle Ages.

The distribution of human stable oxygen isotope values confirms the rich historical and cultural heritage of the sites of Tertiveri and Montecorvino. Our study identified buried individuals potentially originating from the Alpine region and from other Mediterranean locations. However, spatial mobility may have also taken place at smaller geographical scales. In this respect, transhumance activities, mentioned above, would require seasonal movements that are not detectable from bulk isotopic measurements due to a lack of temporal resolution^61^. Late medieval written sources, mainly originating from legislative and administrative records, provide detailed information on regulations applied to transhumance activities, calendar dates for the practice, and rent land prices in Capitanata^58^. Documents indicate that during the cold season (September–May), herds from the Abruzzo Apennines were grazed in the Tavoliere plain in Capitanata.

Migratory movements from the Alps to Capitanata during the late thirteenth century are described in written records^26^. These consisted of military expeditions and the settlement of veterans in the region following the Angevin conquest of the Kingdom of Sicily (1266)^4^. Documents attest that Angevin soldiers were enrolled from Bourgogne and Savoy (south-east France) and detached to the castle of Crepacore in 1269^26^. Once the region was pacified, these soldiers obtained lands nearby Crepacore and likely settled there with their families. A Franco-Provençal dialect similar to those from the French-Swiss border is spoken still today in two communities located less than 15 km from Tertiveri^26^. Most individuals from Cluster B (C92, TC98, TC99, and TC101) were buried in the so-called ‘ordinary’ cemetery outside Tertiveri Cathedral. There are no clear indications that these were high-status individuals (two were assigned to dietary Cluster 1 and two to Cluster 2 and they could be Angevin soldiers or their family members).

There is no precise chronology for the individuals mentioned above but it is known that this differs from other individuals showing oxygen isotopic values associated with the Alpine region (outliers TC20, TC77, TC82, and individual TC83 from Cluster B). These date to the first half of the thirteenth century and were buried in a privileged burial shaft. During the proposed chronology, the Capitanata region was part of the Kingdom of Sicily and Holy Roman Empire, both ruled by Frederik II Hohenstaufen (1194–1250 CE). It is possible that these individuals had their origins in southern Germany and their presence on the site may be related to military expeditions (e.g. the sixth crusade) or diplomatic and political activities.

Individual TC8 was buried according to an Islamic rite and dated to the last decade of the thirteenth century. In 1296 CE, the bishopric was donated by the King Charles II of Naples to ‘Abd al- ‘Azīz, a Muslim knight from the city of Lucera^23^. Lucera was a fortified town in Capitanata where Muslims from Sicily were resettled in the 1220 s by Frederik II following religious unrests on the island^4,23,24^. The Islamic population of Lucera could live according to Muslim laws and cultural habits. They were also heavily involved in trading activities and the wars of the Kingdom. Within this, ‘Abd al- ‘Azīz was one the most influential people and likely was given rule over Tertiveri by King Charles II following his participation in the War of the Vespers (1282–1302) in Sicily^23^. Tertiveri remained under ‘Abd al- ‘Azīz’s control only until 1300 CE, when it was then donated to a Christian knight. Given the privileged burial and the short chronology, individual TC8 is likely a member of ‘Abd al- ‘Azīz’s clan. His oxygen isotopic values indicate that he likely travelled from coastal regions in the western Mediterranean, including Sicily, or from north African coasts. A possibility involves the participation of this individual within the War of the Vespers in Sicily, alongside ‘Abd al- ‘Azīz’.

Future research avenues could be pursued to further the knowledge presented in this paper. In particular, employing techniques such as radiocarbon dating and Bayesian chronological modelling could allow for a more precise chronology, which in turn could offer a more detailed insight into diachronic trends in the socio-economic strategies and links to changing political regimes in Capitanata. The small skeletal collections available for analysis from Montecorvino and San Lorenzo may be augmented by a recently recovered large skeletal assemblage from Montecorvino, which could offer a more comprehensive view of local lifeways in relation to biological sex and age. Additionally, further refinement of food isotopic values through direct analysis of archaeological materials could potentially lead to more precise Bayesian dietary estimates.

## Conclusions

The farming economy of Late medieval Capitanata was based on cereal production and complemented by animal management practices. The rearing of animals, for the most part pigs and ovicaprids, allowed for intensive crop manuring. This practice is apparently absent in other Late medieval sites in southern Italy and may have contributed to a local demographic growth following the establishment of the Norman rule. Predominant cereal production consisted of wheat and barley although millet and/or sorghum were also grown in smaller amounts. The latter may have been linked to transhumance practices. Minor consumption of marine fish, potentially associated to Christian fasting rules, shows the existence of trade routes connecting the sub-Apennine region to the Adriatic coast.

Significant differences in individual diets likely reflect a socioeconomic hierarchy which determined purchase power. Millet and/or sorghum, viewed as lower quality grains, were more affordable than wheat or barley. While animal protein, including marine fish, were costlier food items and more easily accessible to higher status individuals. Among these were likely German elites or Angevin soldiers that moved into the region from Alpine regions during the thirteenth century. One single individual, buried according to an Islamic rite, likely travelled from other western Mediterranean coastal regions (e.g. Sicily).

The traditional image of Late Medieval southern Italy is one where a farming economy sustains a hierarchical feudal system and where a succession of foreign influences conflicted and co-existed. Our multi-isotope Bayesian approach employed in the study of bioarchaeological remains largely corroborates such an image but it also offers a high resolution reconstruction of the socioeconomic, cultural, and demographic dynamics of Late medieval communities. These historical aspects had a formative role that remains visible in the linguistic, religious, culinary, and genetic heritage of southern Italy.

## Methods

We carried out bulk collagen and bioapatite stable carbon, nitrogen and oxygen isotope analyses on skeletal samples from the late medieval sites of Tertiveri, Montecorvino and San Lorenzo in Carminiano^54,69,70,76^. Osteological material was sampled with the permission and collaboration of the Soprintendenza Archeologia, Belle Arti e Paesaggio per le Province di Barletta-Andria-Trani e Foggia. Individuals from Tertiveri are currently stored at the Soprintendenza in Foggia, whereas those from Montecorvino and San Lorenzo are curated by the University of Foggia. A description of the archaeological sites and of a preliminary osteological analysis can be found in Supplementary Information File S2. In total, 134 human individuals and 21 faunal specimens were sampled for stable isotope analysis (Supplementary Information File S1; https://www.doi.org/10.48493/w01v-fe90).

The largest number of human samples was recovered from Tertiveri (113 individuals). This included a so-called ‘bishop’ (TC74) and two individuals buried following an Islamic rite (TC8, TC21). Anthropological analysis from Tertiveri revealed a biological sex ratio of approximately three males to each female. Seven females, ten probable females, fourteen males and twenty-five probable males were identified across the adolescent and adult population. However, it was not possible to determine the biological sex of thirty-seven adolescent and adult individuals due to their fragmented and/or commingled state. The same applied to twenty-one non-adult individuals who did not display clear sexual dimorphism. The site was characterized by a high mortality rate among young adults aged between twenty and forty years old. Also a few elderly individuals over sixty were identified. Non-adults of all age groups were underrepresented, which is a common trait of medieval populations in Italy^77^. Supplementary Information File S2 provides additional detail on biological sex and age at death distributions in Tertiveri.

The dataset from Montecorvino (n = 17) included four adult females, six adult males, and seven non-adults. This represents the original anthropological core of the site, whereas more recently a larger assemblage was excavated. Therefore, demographic profiles are still under investigation and cannot be determined yet. From San Lorenzo (n = 4), two males, one female, and one undetermined adult could be sampled. Further details on this preliminary osteological analysis from the two sites is presented in Supplementary Information File S2.

For comparability purposes we sought to sample rib bones from all individuals. However, many skeletons found in Tertiveri were disarticulated and some did not have preserved ribs. Turnover rates of skeletal bioapatite and collagen vary with osteological element^32^. In the case of ribs, the collagen reflects the c. 3–5 years prior to death of an individual. For many osteological elements their turnover rates are not precisely know nor how these vary with the age of an individual. Thus, our dietary and mobility isotopic comparisons may reflect different life stages of the analysed individuals. The skeletal samples from Tertiveri consisted of: fifty ribs, twenty-nine cranium fragments (mostly parietal and frontal parts), twelve fibulae, four metatarsal bones, four vertebrae, three tibiae, two femora, two humeri, two scapulae, one clavicula, one mandibular fragment, one metacarpal bone, and one radius. In some instances, it was possible to sample multiple tissues (e.g. tooth plus bone) from the same human (e.g. TC86) or animal individual (e.g. MOPI1). In Montecorvino (n = 17) and San Lorenzo in Carminiano (n = 4), human ribs were sampled for all individuals.

The faunal dataset is composed by five specimens from Tertiveri, seventeen from Montecorvino, and one from San Lorenzo. The Tertiveri animal dataset consists of bone samples from two cattle, one pig, one ovicaprid, and one tortoise. From Montecorvino, bone fragments from two cattle, two poultry, one horse, four ovicaprids and one tortoise were collected. In addition, five teeth and two bone fragments from five pigs were also included. From San Lorenzo, only one tooth and one bone from the same deer specimen were sampled. Despite potential turnover differences across bone samples and teeth, we assumed that the shorter lifespan for animals should not significantly impact the interpretation of isotopic results.

Samples from Tertiveri were pre-treated at the stable isotope lab of the Max Planck Institute of Geoanthropology (former “for the Science of Human History in Jena”) (MPI-GEA), Germany, whereas samples from Montecorvino and San Lorenzo in Carminiano were pre-treated at the ‘iCONa’ lab at the Università degli studi della Campania ‘Luigi Vanvitelli’ in Caserta, Italy. All stable isotope measurements were carried out at the MPI-GEA. Sample pretreatment and analytical measurements are described in detail in Supplementary Information File 4.

We employed the Bayesian dietary mixing model ReSources (previously FRUITS) to quantitatively reconstruct late medieval diets in Capitanata^45,55,66,78^. Modelling options are described in Supplementary Information File 3.

Human mobility was investigated using δ^18^O bone measurements. These were compared with a reference baseline for δ^18^O values of drinkable water across western Europe. This baseline relies on a dataset of mean δ^18^O_Water_ annual values from the Regionalized Cluster-based Water Isotope Prediction model (RCWIP)^67^ from which a smoothed spatial surface for δ^18^O was created using the Bayesian model AverageR^45,79^. Probabilistic assignment of residence was generated using the model LocateR^45,68^. For a direct comparison of human bone and water δ^18^O values, the values for the former, reported as carbonate measurements relative to the standard VPDB, were re-expressed relative to the VSMOW standard (δ^18^O_VSMOW_ = δ^18^O_VPDB_ * 1.03092 + 30.92). Following Chenery et al.^80^ carbonate values were converted into phosphate (δ^18^O_phosphate_ = δ^18^O_carbonate_ * 1.0322−9.6849) and finally into drinkable water following Pollard et al.^43^ (δ^18^O_Water_ = δ^18^O_phosphate_ * 1.55−33.49). For modelling purposes and in accordance with previous studies, we employed a conservative 2‰ uncertainty for modelled human δ^18^O_Water_ values which reflects uncertainty introduced by a variety of factors (e.g. cooking, bone diagenesis, etc.)^41,42^.

For cluster classification of human δ^13^C and δ^15^N values we employed the mclust R package^81^. Mixture estimation relied on the expectation–maximisation algorithm and the optimal number of clusters was identified using the Bayesian Information Criterion (BIC). For cluster classification of human δ^18^O values we employed the tclust R package^82^. This gave a cluster assignment similar to mclust but tclust employs a trimming approach^83^, based on a threshold value, to identify outliers. For our application we employed a trimming threshold value of 5%.

### Ethic statement

Archaeological, anthropological, and isotopic analysis of individuals from the sites of Tertiveri, Montecorvino, and San Lorenzo in Carminiano were conducted with the permission and collaboration of the Soprintendenza Archeologia, Belle Arti e Paesaggio per le province di Barletta-Andria-Trani e Foggia.

## Supplementary Information

Supplementary Information 1.

Supplementary Information 2.

Supplementary Information 3.

Supplementary Information 4.

## Data Availability

Data that support the findings of this study are available in Supplementary Information file S1 and at https://www.doi.org/10.48493/w01v-fe90.
