# Supplementary material for: A Bayesian multi-proxy contribution to the socioeconomic, political, and cultural history of late medieval Capitanata (southern Italy)
Source: Sci Rep. 2023 Mar 11;13:4078. doi: 10.1038/s41598-023-30706-9 (PMC10008551; doi:10.1038/s41598-023-30706-9)
Supplement: Supplementary file 2 — Supplementary Information 2. [file 41598_2023_30706_MOESM2_ESM.docx]

**Supplementary Information File 2.**

**S2. Description of the Archaeological Sites and Osteological Analysis**

**S2.1 Tertiveri**

The bishopric of Tertiveri was fortified in the early eleventh century as part of a hilltop defensive system set between Campania and Apulia. The ongoing archaeological excavation of the site involving the Deutsches Historisches Institut (DHI) in Rome and led by the Universität Trier in cooperation with the Soprintendenza Archeologia, Belle Arti e Paesaggio per le Province di Barletta-Andria-Trani e Foggia and other European institutions, has unearthed two churches, traces of a settlement, fortifications, and a residential tower^1–5^. The bishopric was in decline by mid thirteenth century, and in 1296 the site was given by the king of Naples Charles II to ‘Abd al-‘Azīz, a Muslim knight from the nearby city of Lucera^6^. An adult and an infant Islamic individual according to the burial rite, were found in the cemetery, respectively TC8 and TC21^7^. In 1300 Tertiveri was given to a Christian knight.

*S2.1.1 Osteological analysis of the human skeletons from Tertiveri*

In total, 113 human skeletons from Tertiveri were included in this study. Osteological material was sampled with the permission and collaboration of the Soprintendenza Archeologia, Belle Arti e Paesaggio per le Province di Barletta-Andria-Trani e Foggia and this is currently stored at the Soprintendenza in Foggia. The majority of the human dataset was recovered from four burial shafts (features 105, 112, 142, 143) parallel to the external walls of the Cathedral church (Samples TC1-21, 61-77, 79-84, 113)^8^. These burials likely include high-status individuals, among which the so-called ‘Bishop’ (TC74). Neonates, small infants and children are underrepresented in these burial shafts, therefore they were presumably buried in the nearby ‘ordinary’ cemetery (e.g. TC78). Also 26 samples from this ‘ordinary’ cemetery were recovered during fieldwork activities, before the archaeological excavations began, on the NE of the Tertiveri Cathedral (Samples TC86-112). At a second church, 40 mostly incomplete burials were sampled (Samples TC22-60, 85)^9^. Radiocarbon analysis was carried out on skeletons from funeral shaft 105. These date the burials from the eleventh to the thirteenth centuries^7^. The burials contained commingled remains and their ^14^C analysis showed cases of secondary deposition of earlier burials^7^. Congenic pathological features observed in some of the skeletons suggest some relatedness among commingled individuals^7,10^. Within the feature 105, the skeleton of a senile adult (c. 70-80 years old, TC74) was found together with the remains of a pastoral shaft and therefore supposed to belong to a bishop^7,11^.

Prior to sampling for isotopic analysis, skeletal remains were analysed anthropologically and documented (see below). The bone surface preservation of the remains was overall good^12^ and samples taken for isotopic analyses had a high collagen content^13^ (also see Supplementery Information File 1). However, materials recovered from the ‘ordinary’ cemetery during fieldwork activities were heavily fragmented due to recent ploughing and, for most individuals, collagen was not preserved. For conservation purposes, only fragmented bones were taken for isotopic analysis. For this reason, sampled bone material was sometimes barely sufficient for collagen extraction, hence bioapatite stable carbon and oxygen isotope analysis was not carried out for all individuals.

Ribs were preferred for sampling, however, due to the presence of commingled skeletons, these could not be sampled systematically. Whenever ribs were unavailable or not clearly associated with an individual, long bones or skull fragments were sampled. In alternative, whichever bone fragment available and clearly associated to an individual was sampled.

All human remains were studied using well established anthropological methods. A preliminary report of the results of anthropological and palaeopathological analysis was published^7^. This included a case study on a defect of the neural arch^10^ and a case of leprosy.

Biological sex in adult individuals was assessed through the observation of pelvis and cranium^14–16^. Whenever standard methods could not be employed due to limited skeletal completeness and/or preservation, metrical sex determination was used. In this case, femur circumference^17^, and talus and calcaneus^18^ were measured. Furthermore, robusticity was recorded, and sometimes also used for sex assessment.

Age at death in non-adults was mainly assessed using tooth development charts^19,20^ and long bone length^21,22^. For foetuses, Kósa (1978)^23^ was followed. Also the epiphyseal and apophyseal closure of the postcranial bones was observed^15^. In adults, age at death was determined through closure of the cranial sutures, the relief of the pubic symphysis, and the spongiosa structure of the humeral and femoral heads^14–16^. Furthermore, whenever these could not be used, age at death was determined through morphological changes of the sternal rib end^24^, palatal suture closure^25^ and facies auricularis^26^. The definition of measurements of cranial and postcranial bones (e.g. for height estimation) followed Bräuer (1988)^27^  [and Martin (1928)](https://www.zotero.org/google-docs/?XqB54J)^28^. Whenever possible, body heights were also calculated according to Pearson (1898)^29^. These Pearson’s formulas are best adapted for height estimations in ancient and medieval skeletons^30^, and the mathematical and anatomical estimates do not differ significantly from each other^31^. Epigenetic features, and pathological alterations of the cranial area (including teeth), and the postcranial skeleton were also recorded. These will be reported in a forthcoming publication. Biological sex and age at death distribution for each burial ground in Tertiveri is recorded below in Table S2.1.1, S2.1.2, and S2.1.3.

| Age Class | M | F | M=F | Total |
| --- | --- | --- | --- | --- |
| 0-1y |  |  | 0.65 | 0.65 |
| -5y |  |  | 1.85 | 1.85 |
| -10y |  |  | 1.5 | 1.5 |
| -20y |  |  | 0.1 | 0.1 |
| -30y | 8.3 | 2 | 3.5 | 13.8 |
| -40y | 5.5 | 1 | 4.5 | 11 |
| -50y | 0.7 | 1 | 1 | 2.7 |
| -60y | 1.2 |  |  | 1.2 |
| >60y | 1.3 |  |  | 1.3 |
| 21+ |  | 1 | 6.9 | 7.9 |
| 40+ | 1 |  | 2 | 3 |
| Total | 18 | 5 | 22 | 45 |

Tab. S.2.1.1. Sex and age distribution (n) of the sampled individuals in in the Privileged Burial

Shafts (N=45).

| Age Class | M | F | M=F | Total |
| --- | --- | --- | --- | --- |
| 0-1y |  |  | 2.5 | 2.5 |
| -5y |  |  | 3.45 | 3.45 |
| -10y |  |  | 1.05 | 1.05 |
| -20y | 1 | 0.5 | 1 | 2.5 |
| -30y | 6.5 | 5.5 | 1.5 | 13.5 |
| -40y | 6 | 4 | 0.5 | 10.5 |
| -50y | 2.5 |  |  | 2.5 |
| -60y | 1 | 1 |  | 2 |
| >60y | 1 |  |  | 1 |
| 21+ | 1 |  |  | 1 |
| 40+ |  |  |  |  |
| Total | 19 | 12 | 9 | 40 |

Tab. S2.1.2. Sex and age distribution of the sampled individuals in in the Second Church

Cemetery (N=40).

| Age Class | M | F | M=F | Total |
| --- | --- | --- | --- | --- |
| 0-1y |  |  | 1.7 | 1.7 |
| -5y |  |  | 3.8 | 3.8 |
| -10y |  |  | 3.1 | 3.1 |
| -20y | 0.7 |  | 3.4 | 4.1 |
| -30y | 0.3 |  | 2 | 2.3 |
| -40y |  | 0.5 | 2 | 2.5 |
| -50y |  | 0.5 | 1 | 1.5 |
| -60y |  |  | 1 | 1 |
| >60y |  |  |  |  |
| 21+ | 1 | 1 | 6 | 8 |
| 40+ |  |  |  |  |
| Total | 2 | 2 | 24 | 28 |

Tab. S2.1.3. Sex and age distribution of the sampled individuals in in the Cathedral Ordinary

Cemetery (N=28).

In the burial shafts, some animal remains were recovered and five of them were sampled for isotope analysis: Two cattle (TCCA1-2), one ovicaprid (TCSG1), one pig (TCPI1), and one tortoise bone (TCTO1).

**S2.2 Montecorvino and San Lorenzo in Carminiano**

The late medieval site of Montecorvino was founded as part of the same defensive network that included Tertiveri. The archaeological excavation of the site is still ongoing under the leadership of the Università di Foggia in collaboration with the local Soprintendenza office. Previous campaigns focused on the local Cathedral, segments of the settlement, and on fortified architectures^32–36^. Only sporadic traces from the Byzantine period (early eleventh century) are attested whereas most archaeological remains date to the Norman period. Norman-Swabian Montecorvino observed a structural defensive restyling transforming the fortification into a two-tower system (motte and bailey). In addition, the development of a coeval urban centre is also attested. The site presented traces of different productive and crafting activities (e.g. evidence of pottery and lime kilns) and silos, indicating an economy based on cereal production. The decline of the settlement started in the fifteenth century.

San Lorenzo in Carminiano was a late medieval rural settlement located in the Tavoliere plain. The archaeological site has only been partly investigated through archaeological surveys and an excavation led by the Università di Foggia in collaboration with the Soprintendenza office^37–39^. Sporadic traces of an earlier Roman and Late Antiquity occupation have been observed, but the main structure of the settlement appears to date to the late-eleventh century. Most of the local economy appears grounded on crafting activities, animal husbandry - in particular pigs - and on cereal production. A demographic increase is observed in the thirteenth century, as evidenced by the creation of a suburban area. From the fourteenth century onwards there was a progressive decline of the settlement until its abandonment. The main church of San Lorenzo in Carminiano has only been partially excavated.

*S2.2.1 Preliminary assessment of osteological data from Montecorvino and San Lorenzo*

The skeletal assemblage from Montecorvino consists of 17 human individuals and 15 faunal specimens.Osteological material was sampled with the permission and collaboration of the Soprintendenza Archeologia, Belle Arti e Paesaggio per le Province di Barletta-Andria-Trani e Foggia and its study is curated by the University of Foggia (Laboratorio di Archeologia, Dipartimento di Studi Umanistici). Human skeletons were recovered during the excavation of the Cathedral in Montecorvino, but one individual from this assemblage was instead buried in the castle area (i.e. MO17) and dates somewhere between the mid-eleventh to the mid-thirteenth centuries. Graves contained single and multiple individuals and were located along the lateral walls of the building, both externally and internally. Those placed inside the building (e.g. T.1) are assumed to contain wealthier individuals. Some of the individuals from Montecorvino were radiocarbon dated and preliminary results suggest they date from the eleventh to the sixteenth centuries, with the majority dating to the Norman, Staufen, and Angevin periods.

From San Lorenzo only four human individuals could be analysed plus one deer specimen. The latter was sampled for isotopic analysis since there is historical evidence of a royal hunting ground nearby the site^39^ which may have carried out deer management. Human remains were recovered from three graves discovered on the external frontal part of the only church excavated on the site. These graves have been radiocarbon dated to the Norman or Staufen periods (1053-1266).

Completeness^12^, preservation of the human skeletons^12^, and collagen preservation was overall good according to established parameters^13^ (see also Supplementary Information File 1). Analysed individuals presented overall high level of completeness (above 75%). For Montecorvino and San Lorenzo, it was possible to sample rib bones from all individuals for isotopic analysis. Sex identification focused on the two skeletal regions that represent the most reliable sexual dimorphism, i.e. cranium and pelvis. An assessment was carried out through both morphological^14^ and metric^40^ analysis. Age at death was determined via cranial sutures^41,42^, pubic symphysis morphology^43–46^ and facies auricularis^47^. Demographic and palaeopathological analyses are currently undergoing as a larger assemblage was excavated only recently. Faunal specimens were identified following Schmid’s (1972)^48^ methods.

**References**

1. Clemens, L. & Matheus, M. Troia und Tertiveri – Transformationen byzantinischer Bischofssitze in Süditalien. in *Menschen, Bilder, Sprache, Dinge. Wege der Kommunikation zwischen Byzanz und dem Westen. 2 Menschen und Worte* (eds. Daim, F., Gastgeber, C., Heher, D. & Rapp, C.) 225–234 (Verlag des Römisch-Germanischen Zentralmuseums, 2018).

2. Clemens, L. & Muntoni, I. M. New archaeological perceptions to the provençal settlement in the fortress of Lucera and to the fortification of Tertiveri (prov. Foggia). in *Vivre au Château. ‘XXIXe colloque Château Gaillard, Château-Thierry 26 août - 1er septembre 2018’* (eds. Ettel, P., Flambard Héricher, A.-M. & O’Conor, K. D.) vol. 29 77–83 (Presses Universitaires de Caen, 2020).

3. Clemens, L. & Zimmer, J. An architectural survey of the Medieval residential tower at Tertiveri (Foggia province, Apulia). in *Château et commerce* (eds. Ettel, P., Flambard Héricher, A.-M. & O’Conor, K. D.) 91–98 (Presses Universitaires de Caen, 2016).

4. Clemens, L. & Zimmer, J. Bauforschungen am mittelalterlichen Wohnturm von Tertiveri (Prov. Foggia) in Apulien. *Archaeologia Mosellana* **9**, 639–661 (2014).

5. Matheus, M. Lucera, Tertiveri, Bischofsstadt und Bischofssitz. Muslimische Stadt und muslimische Adelsresidenz. Genese eines interdisziplinären Forschungsprojektes zur Geschichte Süditaliens. in *Christen und Muslime in der Capitanata im 13. Jahrhundert. Archäologie und Geschichte* (eds. Clemens, L. & Matheus, M.) vol. 2 109–130 (Kliomedia, 2018).

6. Engl, R. ‘Abd al-‘Azīz von Lucera (gest. 1301). Aufstieg und Fall eines muslimischen Ritters im Königreich Sizilien. in *Christen und Muslime in der Capitanata im 13. Jahrhundert. Archäologie und Geschichte* (eds. Clemens, L. & Matheus, M.) vol. 2 231–249 (Kliomedia, 2018).

7. Teegen, W.-R. Die Toten aus der Bischofsgruft von Tertiveri (Prov. Foggia, Apulien), Ausgrabung 2011. Vorbericht über die anthropologisch-paläopathologischen Untersuchen. in *Christen und Muslime in der Capitanata im 13. Jahrhundert. Archäologie und Geschichte* (eds. Clemens, L. & Matheus, M.) vol. 2 275–286 (Kliomedia, 2018).

8. Clemens, L., Matheus, M., Muntoni, I. M., Pösche, H. & Teegen, W.-R. Nel feudo del vassallo musulmano. *Archeo* vol. 442 82–87 (2021).

9. Clemens, L. & Pösche, H. Eine Bischofsgruft aus Tertiveri (Prov. Foggia). in *Christen und Muslime in der Capitanata im 13. Jahrhundert. Archäologie und Geschichte* (eds. Clemens, L. & Matheus, M.) vol. 2 259–274 (Kliomedia, 2018).

10. Mann, R. W., Burch, A., Barnes, E., Teegen, W.-R. & Chrysostomou, P. T. The Articulating Neural Arch: A Rare Developmental Anomaly. *Forensic Anthropology* **1**, 180–186 (2018).

11. Gianandrea, M. Signa Christiana a Tertiveri: un inedito riccio di pastorale. in *Christen und Muslime in der Capitanata im 13. Jahrhundert. Archäologie und Geschichte* (eds. Clemens, L. & Matheus, M.) vol. 2 287–295 (Kliomedia, 2018).

12. Nováček, J., Scheelen-Nováček, K., Schultz, M., Bjørnstad, G. & Steskal, M. *Das Grabhaus 1/08 in der Hafennekropole von Ephesos*. *ISBN* vol. XVI/1 9–10 (Verlag der Österreichischen Akademie der Wissenschaften, 2020).

13. Ambrose, S. H. Preparation and characterization of bone and tooth collagen for isotopic analysis. *Journal of Archaeological Science* **17**, 431–451 (1990).

14. Acsádi, G. & Neméskeri, J. *History of human life span and mortality*. (Akademiai Kiado, 1970).

15. Ferembach, D., Schwindezky, I. & Stoukal, M. Recommendations for age and sex diagnosis of skeletons. *Journal of human evolution* **9**, 517–549 (1980).

16. Rösing, F. W. *et al.* Recommendations for the forensic diagnosis of sex and age from skeletons. *HOMO* **58**, 75–89 (2007).

17. Black III, T. K. A new method for assessing the sex of fragmentary skeletal remains: Femoral shaft circumference. *American Journal of Physical Anthropology* **48**, 227–231 (1978).

18. Steele, D. G. The estimation of sex on the basis of the talus and calcaneus. *Am J Phys Anthropol* **45**, 581–588 (1976).

19. Ubelaker, D. H. *Human Skeletal Remains: Excavation, Analysis, Interpretation*. (Aldine De Gruyter, 2008).

20. Brothwell, D. R. *Digging Up Bones*. (Cornell Univ Pr, 1981).

21. Scheuer, L. & Black, S. M. *Developmental juvenile osteology*. (Academic Press, 2000).

22. Scheuer, L., Black, S. & Schaefer, M. C. *Juvenile Osteology: A Laboratory and Field Manual*. (Academic Press, 2008).

23. Kósa, F. Identifikation des Feten durch Skelettuntersuchungen. in *Identifikation* (eds. Hunger, H. & Leopold, D.) 211–241 (J. Barth, 1978).

24. İşcan, M. Y. & Loth, S. R. Estimation of age and determination of sex from the sternal rib. in *Forensic Osteology: Advances in the Identification of Human Remains* (eds. Reichs, K. J. & Bass, W. M.) 68–89 (Charles C. Thomas, 1998).

25. Mann, R. W., Jantz, R. L., Bass, W. M. & Willey, P. S. Maxillary suture obliteration: a visual method for estimating skeletal age. *J Forensic Sci* **36**, 781–791 (1991).

26. Buckberry, J. L. & Chamberlain, A. T. Age estimation from the auricular surface of the ilium: a revised method. *Am J Phys Anthropol* **119**, 231–239 (2002).

27. Bräuer, G. Osteometrie. in *Anthropologie: Handbuch der vergleichenden Biologie des Menschen 1* (eds. Knussmann, R. & Martin, R.) vol. 1 160–232 (Gustav Fischer, 1988).

28. Martin, R. *Lehrbuch der Anthropologie in systematischer Darstellung: Kraniologie, Osteologie*. vol. 2 (Gustav Fischer, 1928).

29. Pearson, K. Mathematical contributions to the theory of evolution. V. On the reconstruction of the stature of prehistoric races. *Proceedings of the Royal Society of London* **63**, 417–420 (1898).

30. Siegmund, F. *Die Körpergröße der Menschen in der Ur- und Frühgeschichte Mitteleuropas und ein Vergleich ihrer anthropologischen Schätzmethoden*. (Books on Demand, 2010).

31. Sierp, I. & Henneberg, M. Reconstruction of body height from the skeleton: Testing a dozen different methods for consistency of their results. *Anthropologischer Anzeiger* **73**, (2016).

32. Favia, P., Giuliani, R. & De Venuto, G. La ricerca archeologica sul sito di Montecorvino: le campagne di scavo 2009-2010. in *Atti del 32° Convegno nazionale sulla Preistoria - Protostoria - Storia della Daunia. San Severo 12-13 novembre 2011* (ed. Gravina, A.) 331–354 (Archeoclub San Severo, 2012).

33. Favia, P. *et al.* La ricerca archeologica sul sito di Montecorvino. Le campagne di scavo 2011-2014. in *Atti del 35° Convegno Nazionale sulla Preistoria - Protostoria - Storia della Daunia. San Severo, 15-16 novembre 2014* (ed. Gravina, A.) vol. 1 141–164 (Archeoclub di San Severo, 2015).

34. Favia, P. *et al.* Montecorvino: Parabola insediativa di una cittadina dei Monti Dauni fra XI e XVI secolo. in *VII Congresso nazionale di archeologia medievale. ‘Palazzo Turrisi, Lecce, 9-12 settembre 2015’* (eds. Arthur, P. & Imperiale, M. L.) 191–196 (All’insegna del giglio, 2015).

35. Favia, P. *et al.* Modelli di trattamento degli alimenti in un contesto castrense medievale: la cucina e la dispensa della rocca di Montecorvino. *Facta. A Journal of Late Roman, Medieval and Post-Medieval Material Culture Studies* **8**, 25–56 (2014).

36. Giuliani, R. & Favia, P. La ‘Sedia del Diavolo’. Analisi preliminare delle architetture del sito medievale di Montecorvino (Foggia) in Capitanata. *Archeologia dell’Architettura* **7**, 133–159 (2007).

37. Caracuta, V., Fiorentino, G. & Corvino, C. Ambiente e strategie produttive nei siti di San Lorenzo in Carminiano e Pantano (Fg) tra XIII e XIV secolo. in *Federico II e i cavalieri teutonici in Capitanata: recenti ricerche storiche e archeologiche. ‘Atti del Convegno Internazionale (Foggia-Lucera-Pietramontecorvino, 10-13 giugno 2009)’* (eds. Favia, P., Houben, H. & Toomaspoeg, K.) 317–332 (Congedo, 2012).

38. De Venuto, G. *Allevamento, ambiente ed alimentazione nella Capitanata medievale: archeozoologia e archeologia globale dei paesaggi*. (Edipuglia, 2013).

39. Favia, P. *et al.* San Lorenzo “in Carminiano” presso Foggia: indagine archeologica su un sito medievale del Tavoliere di Puglia in un contesto di moderna espansione edilizia. in *Atti del V Congresso nazionale di archeologia medievale. Foggia-Manfredonia 30 settembre-3 ottobre 2009* (eds. Volpe, G. & Favia, P.) 382–391 (All’insegna del giglio, 2009).

40. Pettener, D. & Brasili Gualandi, P. La funzione discriminante nella diagnosi del sesso in base ai caratteri metrici del femore. *Antropologia Contemporanea* **2**, 59–68 (1979).

41. Meindl, R. S. & Lovejoy, C. O. Ectocranial suture closure: A revised method for the determination of skeletal age at death based on the lateral-anterior sutures. *American Journal of Physical Anthropology* **68**, 57–66 (1985).

42. Nemeskéri, J., Harsányi, L. & Acsádi, G. Methoden zur diagnose des lebensalters von skelettfunden. *Anthropologischer Anzeiger* 70–95 (1960).

43. Brooks, S. & Suchey, J. M. Skeletal age determination based on the os pubis: A comparison of the Acsádi-Nemeskéri and Suchey-Brooks methods. *Human Evolution* **5**, 227–238 (1990).

44. Burns, K. R. *Forensic anthropology training manual*. (Routledge, 2015).

45. Katz, D. & Suchey, J. M. Age determination of the male Os pubis. *American Journal of Physical Anthropology* **69**, 427–435 (1986).

46. Todd, T. W. Age changes in the pubic bone. *American journal of physical anthropology* **4**, 1–70 (1921).

47. Lovejoy, C. O., Meindl, R. S., Pryzbeck, T. R. & Mensforth, R. P. Chronological metamorphosis of the auricular surface of the ilium: A new method for the determination of adult skeletal age at death. *American Journal of Physical Anthropology* **68**, 15–28 (1985).

48. Schmid, E. *Atlas of animal bones. For prehistorians, archaeologists and Quaternary geologists. Knochenatlas. Für Prähistoriker, Archäologen und Quartärgeologen*. (Elsevier Pub. Co., 1972).
