# Supplementary material for: A Bayesian multi-proxy contribution to the socioeconomic, political, and cultural history of late medieval Capitanata (southern Italy)
Source: Sci Rep. 2023 Mar 11;13:4078. doi: 10.1038/s41598-023-30706-9 (PMC10008551; doi:10.1038/s41598-023-30706-9)
Supplement: Supplementary file 3 — Supplementary Information 3. [file 41598_2023_30706_MOESM3_ESM.docx]

**Supplementary Information File 3.**

**ReSources parameter and prior selections**

ReSources is an R application used to implement Bayesian mixing models for isotope-based dietary reconstruction^1^. It is an upgraded version of the Bayesian software FRUITS^2^. To improve the precision of dietary estimates, ReSources can also implement prior constraints, based on non-isotopic historical and archaeological dietary evidence (e.g. study of archaeobotanical or zooarchaeological remains, written documents, etc.). The app code is available at GitHub (<https://github.com/Pandora-IsoMemo/resources>) and it can also be run online at https://isomemoapp.com/app/resources. ReSources was used to reconstruct human diets for the late medieval sites of Tertiveri, Montecorvino and San Lorenzo in Carminiano (Apulia, southern Italy).

For dietary modelling we considered seven main food groups which were common in late medieval southern Italy^3^: C_3_ plants, C_4_ cereals, Cattle, Sheep/Goat, Pig, Poultry, and Marine Resources. Freshwater resources were not considered for the model, given that there are no archaeological or historical indications that local freshwater streams could provide for significant amount of foodstuff^4^. Moreover, freshwater resources typically present ^13^C-depleted values^5^ that are not observed in our isotopic results. Estimates of the caloric contributions from these food groups were generated independently for adult population isotopic means (δ^13^C_Collagen_, δ^15^N_Collagen_, and δ^13^C_Carbonate_) for Tertiveri (clusters 1 and 2 separately), San Lorenzo in Carminiano, and Montecorvino.

The food isotopic references for terrestrial fauna relied on available data from Tertiveri and Montecorvino. For other food sources, we relied on previously published values. For marine foods, we relied on isotopic data for Mediterranean fish from the medieval period published by Gismondi *et al.* (2020)^6^. Reference isotopic values for C_3_ plants were obtained from the same publication, as these are the geographically closest available measurements for medieval southern Italy. There was no available isotopic data for medieval C_4_ cereals and we relied on values reported for Bronze Age Greece by Nitsch *et al.* (2017)^7^. Albeit these belong to a different chronological horizon, Greece presents a temperate Mediterranean climate and a similar environment to southern Italy. δ^13^C and δ^15^N values for food remains are listed below in table S3.1.

|  | δ^13^C | δ ^δ15^N |
| --- | --- | --- |
| Charred C_3_ Plant remains | -22.6±0.9‰ | 4.1±1.6‰ |
| Charred C_4_ Cereals remains | -10.4±0.3‰ | 6.8±2.7‰ |
| Cattle bone collagen | -20.7±1.2‰ | 6.3±1.6‰ |
| Ovicaprid bone collagen | -21.6±0.9‰ | 5.7±2.0‰ |
| Pig bone/dentine collagen | -21.9±0.9‰ | 7.1±1.4‰ |
| Poultry bone collagen | -21.1±0.7‰ | 9.5±0.9‰ |
| Marine resources bone collagen | -10.7±3.3‰ | 11±1.7‰ |

Tab. S3.1. Isotopic values for food remains employed in Bayesian dietary modelling. These do not include corrections for offsets between edible tissues and food remains (e.g. muscle meat protein or lipids vs. bone collagen collagen). Corrected values are given in table S3.2.

It is necessary to account for potential differences between the isotopic values measured on food remains and the actual edible food component. For this we employed known offsets to calculate the isotopic values of food macronutrients (protein *versus* lipids/carbohydrates) from food remains. The following offset corrections were then applied, with uncertainties for macronutrient isotopic values rounded up to multiples of 0.5‰ (these are based on Fernandes et al. 2015^5^ and include on update by Soncin *et al.* 2021^8^): Plants: Δ^13^C_protein-bulk_=-2‰, Δ^13^C_carbohydrates-bulk_=+0.5‰, Δ^15^N_protein-bulk_=0‰; terrestrial animals: Δ^13^C_protein-collagen_= -2‰, Δ^13^C_lipids-collagen_= -8‰, Δ^15^N_protein-collagen_=0‰; aquatic animals: Δ^13^C_protein-collagen_=-1‰, Δ^13^C_lipids-collagen_=-7‰, Δ^15^N_protein-collagen_=+1.5‰). Corrected values for each food source are reported below in table S3.2.

|  | δ_13_C_collagen_ Protein | δ_13_C_collagen_ Lipids/Carbohydrates | δ_15_N  Protein | δ_13_C_carbonate_  ‘Bulk’ |
| --- | --- | --- | --- | --- |
| C_3_ Plants | -24.6±2‰ | -22.1±2‰ | 4.1±3‰ | -22.5±3‰ |
| C_4_ Cereals | -12.4±1.5‰ | -9.9±1.5‰ | 6.8±4.0‰ | -10.2±2.5‰ |
| Cattle | -22.7±2.5‰ | -28.7±2.5‰ | 6.3±3‰ | -26.9±3.5‰ |
| Ovicaprid | -23.6±2‰ | -29.6±2‰ | 5.7±3‰ | -27.8±3‰ |
| Pig | -23.9±2‰ | -29.9±2‰ | 7.1±2.5‰ | -28.1±3‰ |
| Poultry | -23.1±2‰ | -29.1±2‰ | 9.5±2‰ | -27.3±3‰ |
| Marine Sources | -11.7±4.5‰ | -17.7±4.5‰ | 12.5±3‰ | -13.8±5.5‰ |

Tab.S3.2. Corrected isotopic food macronutrients values as employed in dietary modelling.

Macronutrient caloric concentration values were reported in Fernandes *et al.* (2015)^5^ but with doubled uncertainty values: Plants (C_4_ cereals): protein: 10±5%; Lipids/Carbohydrates 90±5%, terrestrial animals: protein: 30±5%; Lipids/Carbohydrates 70±5%, and marine resources: protein: 65±10%; Lipids/Carbohydrates 35±10%. In the case of C_3_ plants, we considered the inclusion of pulses and set a higher protein concentration (protein: 15±10%; Lipids/Carbohydrates: 85±10%). The use of human δ^13^C_carbonate_ values for dietary modelling required the inclusion of a fictitious ‘Bulk’ food fraction since that δ^13^C_carbonate_ reflects a dietary carbon mix^9^. In the case of plant remains the bulk value is measured directly. However, for animal food groups a mass balance calculation was done to estimate the bulk values from the mean concentration values for macronutrients (Table. S3.2) and respective isotopic values (Table. S3.1).

Isotopic offsets between diet and human tissues plus dietary routing mechanisms were included in the Bayesian mixing model. Bone collagen δ^15^N was assumed to derive entirely from dietary protein with an isotopic offset of 5.5±0.5‰^5^. For bone collagen δ^13^C we set an offset of 4.8±0.5‰ towards food values^9^. Moreover, we considered that the isotopic signal was routed: 74±4% from dietary protein and 26±4% from lipids/carbohydrates^9^. For bone δ^13^C_carbonate_, we employed an offset of 10.1±0.5‰ and the signal was considered to derive entirely from the ‘Bulk’ component^9^.

To improve the resolution of dietary estimates we employed prior constraints^1,10^ by relying on non-isotopic dietary evidence. Following Ruas (2012)^11^ and Favia *et al.* (2014)^12^ we assumed that C_3_ plants were consumed in larger amounts than C_4_ plants. We also considered that during the Middle Ages, starches were the staple food for most southern Italian populations^3^ and therefore we set the following prior information: C_3_ plants + C_4_ cereals > 57% of the caloric contribution. This percentage was calculated using as a reference the mean caloric contribution from starches consumed in Mediterranean countries between 1960–1965 (table 14.2 reported in Garnsey & Scheidel 1998^13^). Furthermore, using the same principle, we assumed that combined animal sources were less consumed than starches.

Zooarchaeological studies for our research region^12,14–20^ show pigs were more consumed than ovicaprids. Cattle bones were overall less represented than the previous taxa and this is likely an indication that meat from this animal was rarely consumed. No indication is given on cow milk and dairy products, but it can be assumed that ovicaprid milk was considered safer^21^. Poultry is also less represented in the archaeofaunal record and we assumed that their consumption was lower than that of pigs or ovicaprid.

Dietary modelling results are displayed in Fig. 7 in the body of the article. Model inputs used for each population group are available as separate files that can be loaded into the ReSources app:

Tertiveri Cluster 1 and 2: <https://pandoradata.earth/dataset/249a8684-7bee-4508-9a88-ed30391d596a/resource/a87a2de9-77e0-452a-a539-7e36e33cff5f/download/tertiveri-clusters.zip>

Montecorvino and San Lorenzo: <https://pandoradata.earth/dataset/249a8684-7bee-4508-9a88-ed30391d596a/resource/50cf7d52-aca9-406c-9dc8-dad1f4a36b24/download/montecorvino-and-san-lorenzo-model.zip>

**References**

1. Sołtysiak, A. & Fernandes, R. Much ado about nothing: assessing the impact of the 4.2 kya event on human subsistence patterns in northern Mesopotamia using stable isotope analysis. *Antiquity* **95**, 1145–1160 (2021).

2. Fernandes, R., Millard, A. R., Brabec, M., Nadeau, M.-J. & Grootes, P. Food Reconstruction Using Isotopic Transferred Signals (FRUITS): A Bayesian Model for Diet Reconstruction. *PLoS ONE* **9**, e87436 (2014).

3. Montanari, M. *Medieval Tastes. Food, Cooking, and the Table*. (Columbia Univ Press, 2012).

4. Battafarano, M. & De Grossi Mazzorin, J. Analisi dei resti itici da alcuni contesti archeologici della Puglia di età tardoantica e medievale. in *Atti del 5° Convegno Nazionale di Archeozoologia, Rovereto, 10-12 Novembre 2006* 289–292 (Pubblicazione del Museo Civico di Rovereto, 2010).

5. Fernandes, R., Grootes, P., Nadeau, M.-J. & Nehlich, O. Quantitative diet reconstruction of a Neolithic population using a Bayesian mixing model (FRUITS): The case study of Ostorf (Germany). *American Journal of Physical Anthropology* **158**, 325–340 (2015).

6. Gismondi, A. *et al.* A multidisciplinary approach for investigating dietary and medicinal habits of the Medieval population of Santa Severa (7th-15th centuries, Rome, Italy). *PLoS ONE* **15**, e0227433 (2020).

7. Nitsch, E. *et al.* A bottom-up view of food surplus: using stable carbon and nitrogen isotope analysis to investigate agricultural strategies and diet at Bronze Age Archontiko and Thessaloniki Toumba, northern Greece. *World Archaeology* **49**, 105–137 (2017).

8. Soncin, S. *et al.* High-resolution dietary reconstruction of victims of the 79 CE Vesuvius eruption at Herculaneum by compound-specific isotope analysis. *Science Advances* (2021) doi:10.1126/sciadv.abg5791.

9. Fernandes, R., Nadeau, M.-J. & Grootes, P. M. Macronutrient-based model for dietary carbon routing in bone collagen and bioapatite. *Archaeol Anthropol Sci* **4**, 291–301 (2012).

10. Cocozza, C., Cirelli, E., Groß, M., Teegen, W.-R. & Fernandes, R. Presenting the Compendium Isotoporum Medii Aevi, a Multi-Isotope Database for Medieval Europe. *Sci Data* **9**, 354 (2022).

11. Ruas, M.-P. Cultures et moissons à Fiorentino : étude des semences carbonisées. in *Fiorentino ville désertée nel contesto della Capitanata medievale (Ricerche 1982-1993)* (eds. Calò Mariani, M. S., Piponnier, F., Beck, P. & Laganara, C.) 541–565 (École Française de Rome, 2012). doi:10.1400/209868.

12. Favia, P. *et al.* Modelli di trattamento degli alimenti in un contesto castrense medievale: la cucina e la dispensa della rocca di Montecorvino. *Facta. A Journal of Late Roman, Medieval and Post-Medieval Material Culture Studies* **8**, 25–56 (2014).

13. Garnsey, P. & Scheidel, W. *Cities, Peasants and Food in Classical Antiquity: Essays in Social and Economic History*. (Cambridge University Press, 1998).

14. Buglione, A. & De Venuto, G. Lo sfruttamento della risorsa animale in Puglia tra il X e lʼXI secolo: il contributo della ricerca archeozoologica. in *La Capitanata e l’Italia meridionale nel secolo XI da Bisanzio ai Normanni* (eds. Favia, P. & De Venuto, G.) 279–285 (Edipuglia, 2011).

15. De Venuto, G. *Allevamento, ambiente ed alimentazione nella Capitanata medievale: archeozoologia e archeologia globale dei paesaggi*. (Edipuglia, 2013).

16. De Venuto, G. Carni, lane e pellame nell’Italia del medio e basso versante adriatico, tra X e XV secolo. *Post Classical Archaeologies* **3**, 117–138 (2013).

17. De Venuto, G. Contributo alla ricostruzione dei caratteri dell’allevamento transumante ovino tra Abruzzo e Tavoliere di Puglia in età medievale. in *Vie degli Animali, Vie degli Uomini. Transumanza ed altri Spostamenti di Animali nell’Europa Tardoantica e Medievale* (eds. Volpe, G., Buglione, A. & De Venuto, G.) 69–81 (Edipuglia, 2010).

18. De Venuto, G. Forme dell’allevamento suino in Puglia in età medievale: il dato archeozoologico. in *Atti del 5° Convegno Nazionale di Archeozoologia, Rovereto, 10-12 Novembre 2006* (ed. Tagliacozzo, A.) (Pubblicazione del Museo Civico di Rovereto, 2010).

19. De Venuto, G. To Eat and to Be in Medieval Southern Italy: The Zooarchaeological Contribution from Religious, Rural and Fortified Sites. in *Bestial Mirrors. Using Animals to Construct Human Identities in Medieval Europe* (eds. Pluskowski, A., Kunst, G.-K., Kucera, M., Bietak, M. & Hein, I.) 55–61 (VIAS, 2010).

20. De Venuto, G. Animals and Economic Patterns in Medieval Apulia (South Italy): Preliminary Findings. in *Breaking and shaping beastly bodies: animals as material culture in the Middle Ages* (ed. Pluskowski, A.) 217–234 (Oxbow, 2007).

21. Angulo, F. J., LeJeune, J. T. & Rajala-Schultz, P. J. Unpasteurized Milk: A Continued Public Health Threat. *Clinical Infectious Diseases* **48**, 93–100 (2009).
