# Supplementary material for: A Bayesian multi-proxy contribution to the socioeconomic, political, and cultural history of late medieval Capitanata (southern Italy)
Source: Sci Rep. 2023 Mar 11;13:4078. doi: 10.1038/s41598-023-30706-9 (PMC10008551; doi:10.1038/s41598-023-30706-9)
Supplement: Supplementary file 4 — Supplementary Information 4. [file 41598_2023_30706_MOESM4_ESM.docx]

**Supplementary Information File 4.**

**Stable isotope pretreatment protocol and measurements**

Soil was removed from osteological samples using fine sandblasting. Collagen was extracted using a modified Longin (1971)^1^ method. Circa 500mg of bone fragments or tooth dentine were immersed in 10mL of 0.5M HCl acid and stored at 5°C until demineralisation. Once this was complete, samples were rinsed three times using ultra-pure water. The demineralized sample was covered in 10ml of pH=3 solution and placed on a heating block at 70°C for 48 hours. Subsequently, gelatinised collagen samples were filtered using an ezee-filter, frozen for circa 24 hours at -20°C, and then freeze-dried. Duplicate samples of collagen (0.8–1.2mg) were weighed into tin capsules. These were isotopically analysed using a Thermo Scientific Flash 2000 Organic Elemental Analyser coupled to a Delta V Advantage Isotope Ratio Mass Spectrometer. Measurement of samples were interspersed with measurements of international standards (IAEA-N2 (δ^15^N= 20.3), IAEA-CH6 (δ^13^C= -10.49), USGS40 L-Glutamic Acid (δ^13^C= -26, δ^15^N= -4.5)) plus an in-house fish gel standard (δ^13^C= -15.7, δ^15^N=14.3). The overall uncertainties on the measurements of each sample were calculated by repeated measurements of samples and reference materials. Uncertainties are expressed as one standard deviation. The maximum uncertainty for all samples across all runs was 0.28‰ for carbon and 0.16‰ for nitrogen stable isotopes. Isotopic results are reported using delta notation relative to the international standards VPDB and AIR for carbon (δ^13^C) and nitrogen (δ^15^N) stable isotopes, respectively.

Bioapatite and enamel was pretreated following Fernandes *et al.* (2014)^2^ protocol. Approximately 10mg of enamel or bone powder were drilled from the bone/enamel surface. Samples were weighed and then transferred to a micro-centrifuge tube. Approximately 1mL of 0.1M acetic acid was added to samples for ten minutes and vortexed to improve the rate of acid reaction. Samples were then centrifuged and the supernatant removed. Following this, samples were rinsed three times with ultra-pure water. Sample tubes were covered using parafilm and frozen for circa 24 hours at -20°C. Samples were then freeze-dried for circa four hours. Roughly 3.0mg of sample was weighed into borosilicate glass vials. Vials were flush/filled with helium at 100ml/min for 10-min. 100% phosphoric acid was then added to each sample. Gases evolved from the samples were analysed for stable carbon and oxygen isotopic composition using a Thermo Gas Bench 2 connected to a Thermo Delta V Advantage Mass Spectrometer. δ^13^C and δ^18^O values were compared against International Standards (IAEA-603 δ^13^C= +2.46‰, δ^18^O= -2.37‰), IAEA-CO-8 (δ^13^C= -5.8‰; δ^18^O= -22.7‰), IAEA NBS 18 (δ^13^C= -5.014‰, δ^18^O= -23.2‰), and USGS44 (δ^13^C= -42.2‰,). Replicate precision of the standards was used to determine machine measurement error, with a standard deviation of c. ±0.30‰ for δ^13^C and ±0.30‰ for δ^18^O. Overall measurement precision was determined through the measurement of repeat extracts from a horse tooth enamel standard (n= 20, ±0.2‰ for δ^13^C and ±0.3‰ for δ^18^O).

**References**

1. Longin, R. New Method of Collagen Extraction for Radiocarbon Dating. *Nature* **230**, 241–242 (1971).

2. Fernandes, R., Nadeau, M. J. & Grootes, P. M. EDTA based protocols for the cleaning of ancient bone bioapatite. in *Proceedings of the 39th International Symposium for Archaeometry* 73–78 (2014).
